# Micron-Sized Pored Membranes Based on Polyvinylidene Difluoride Hexafluoropropylene Prepared by Phase Inversion Techniques

**DOI:** 10.3390/polym9100489

**Published:** 2017-10-06

**Authors:** Andreas Hofmann, Eva Thißen, Matthias Migeot, Nicole Bohn, Stefan Dietrich, Thomas Hanemann

**Affiliations:** 1 Werkstoffkunde (IAM-WK), Institut für Angewandte Materialien, Karlsruher Institut für Technologie (KIT), Hermann-von-Helmholtz-Platz 1, 76344 Eggenstein-Leopoldshafen, Germany; eva_thissen@web.de (E.T.); matthiasmigeot@hotmail.de (M.M.); stefan.dietrich@kit.edu (S.D.); thomas.hanemann@kit.edu (T.H.); 2 Keramische Werkstoffe und Technologien (IAM-KWT), Institut für Angewandte Materialien, Karlsruher Institut für Technologie (KIT), Hermann-von-Helmholtz-Platz 1, 76344 Eggenstein-Leopoldshafen, Germany; nicole.bohn@kit.edu; 3 Institut für Mikrosystemtechnik, Universität Freiburg, Georges-Köhler-Allee 102, 79110 Freiburg, Germany

**Keywords:** PVdF-HFP, micron-sized pored membrane, phase inversion, gel polymer electrolyte

## Abstract

In this study, micron-sized pored membranes, based on the co-polymer polyvinylidene difluoride hexafluoropropylene (PVdF-HFP) were prepared via phase inversion techniques. The aim of the approach was to find less harmful and less toxic solvents to fabricate such films. Therefore, the Hansen solubility approach was used to identify safer and less toxic organic solvents for the phase inversion process, relative to present solvent mixtures, based on acetone, dimethyl formamide, dimethyl acetamide or methanol. With this approach, it was possible to identify cyclopentanone, ethylene glycol and benzyl alcohol as suitable solvents for the membrane preparation process. Physicochemical and mechanical properties were analyzed and compared, which revealed a uniform membrane structure through the cross section. Differences were observed at the top surface, in dependence of both preparation approaches, which are described in detail.

## 1. Introduction

Micron-sized pored membranes play crucial roles in an enormous variety of different applications and research fields, such as energy storage, catalysis, separation, osmosis, textiles, functional materials and so on [1,2,3,4,5,6,7]. Different techniques can be utilized to produce such membranes in an appropriate manner. In the well-known two-step Bellcore process, a micro-porous structure is formed by a plasticizer, which is removed in a subsequent step [8,9]. However, harmful and highly flammable compounds are used for this procedure, like the solvent diethyl ether or the plasticizer dibutyl phthalate. Gel polymer electrolytes can be prepared directly with electrolyte solvents and conducting salts. Unfortunately, often a low-boiling solvent also has to be used, to ensure a suitable processability, which has to be extracted prior to use [10,11,12]. Another strategy for membrane preparation is using a phase separation process [6,13,14,15,16,17,18]. However, usually, flammable and highly toxic chemicals are employed within these fabrication processes, like acetone [4,6,14,15,16,18,19,20], methanol [14,19], dimethyl acetamide (DMA) [17,21], dimethyl formamide [5,15,22] or *N*-methyl-2-pyrrolidone (NMP) [23]. Although these solvents can be used to prepare high-quality membranes, disadvantageous solvent properties such as toxicity, flammability and vapor pressure often limit their industrial application, due to additional costs. Additionally, parameters have to be adjusted very precisely to give uniform and reproducible membranes. In particular, the use of acetone is critical for large-scale processes, due to the very high vapor pressures and the rapid evaporation. Moreover, often only very few solvents are available for the manufacturing process, based on solubilities and chemical compatibilities. These factors hamper the usage of this process for large-scale industrial applications. Therefore, alternative preparation processes and solvent formulations, based on the phase inversion approach, have been intensively explored. 

The Hansen solubility approach, which shows that the total cohesion energy (*E*) can be used to predict the solubility behaviors of polymers in solvents and solvent blends [24]. Basically, the solubility parameter (*δ*, namely *δ*^2^ = *E*/*V*_m_, where *V*_m_ = molar volume; *E* = cohesive energy), can be divided into three components, according to dispersion forces (*δ*_D_), permanent dipole–dipole forces (*δ*_P_) and hydrogen bonding (*δ*_H_), in order for a description within Cartesian coordinates to be possible. Finally, the solubility parameter distance (*R*_a_, also called HSP Distance) between substances 1 and 2 in the Hansen space can be calculated, as follows (Equation (1)) [24].

(1) $${\left(R_{a}\right)}^{2}=\;4\cdot {\left(\delta_{D2}-\delta_{D1}\right)}^{2}+{\left(\delta_{P2}-\delta_{P1}\right)}^{2}+{\left(\delta_{H2}-\delta_{H1}\right)}^{2}$$

Based on the experimental determination of “good” and “bad” solvents, it is possible to define a sphere, of radius (*R*_0_), for a region in the Hansen space which contains most of the “good” solvents. Subsequently it is possible to calculate the relative energy difference (RED = (*R*_a_)/(*R*_0_)), which predicts good solvents (RED < 1) and bad solvents (RED > 1).

In this study, a novel phase inversion approach, based on less harmful chemicals, is presented, which can be used to prepare micron-sized pored membranes. The intention of the preparation process is to develop a safer approach for preparing micron-sized pored membranes via phase inversion techniques, by avoiding highly toxic (e.g., NMP, dimethylacetamide, dimethylformamide) and flammable (e.g., acetone) solvents [6,14,15,16,17,18,23,25]. Based on the Hansen solubility approach, different solvent mixtures were investigated for the phase inversion process. Two different approaches were investigated in detail, in which temperature is used for the phase inversion process. 

## 2. Materials and Methods

Membrane preparation. Two techniques were used to prepare micron-sized pored membranes, labeled as M-1 and M-2. For the preparation of the membranes, polyvinylidene difluoride hexafluoropropylene (PVdF-HFP) (Solvay; Solef 21216/1001; *M* ~ 400,000 g/mol; M-1: 10 wt %; M-2: 11 wt %) was dissolved in cyclopentanone (TCI Chemicals, Eschborn, Germany; >99%; M-1: 72 wt %; M-2: 56 wt %). Subsequently, ethylene glycol (Sigma-Aldrich, Munich, Germany; >99.5%; 18 wt %; M-1) or benzyl alcohol (TCI Chemicals, Eschborn, Germany; >99%; 33 wt %; M-2) was added and both mixtures were placed on top of a heating plate at 80 °C (M-1) or 90 °C (M-2) for 5 days. For membrane M-1, the mixture (80 °C) was applied by doctoral blade (600 µm gap width) onto a stainless steel plate (room temperature). The plate was placed in a pre-heated oven (55 °C) for 45 min and afterwards onto a heating plate (IKA, C-MAG HP 10, Staufen, Germany) at 80 °C (2 h) and then at 90 °C (15 h). Thereafter, the membrane was placed into iso-propanol (VWR; HiPerSolv, Darmstadt, Germany) for solvent extraction (24 h with shaking). After drying at 80–90 °C onto a heating plate, a white membrane became available. For membrane M-2, the homogenous viscous mixture (90 °C) was applied by doctor blade (600 µm; pre-heated at 70 °C) onto stainless steel, at 70 °C, and allowed to cool down on the laboratory bench. At 50–55 °C the mixture became solid and opaque. After cooling to room temperature, the membranes were placed into iso-propanol (VWR; HiPerSolv, Darmstadt, Germany) to remove both solvents. After 4 h, the wet membrane was placed onto stainless steel and dried at 25 °C (30 min) and then at 80 °C (24 h). White membrane sheets were obtained from this procedure. 

Hansen solubility. In this study, the solubility of PVdF-HFP was investigated, according to the Hansen solubility approach. The results of all experimental solubility tests are listed in the Supplementary Materials (Table S1), in which solvents and non-solvents are distinguished (solvent: a gel-like or liquid behavior is obtained by mixing an organic solvent (4.5 g) and PVdF-HFP (0.5 g); non-solvent: no alteration of the PVdF-HFP (0.5 g) powder is observed within 24 h at 25–50 °C, during contact with 4.5 g organic solvent). 

SEM analysis. A Zeiss Supra 55 SEM scanning electron microscope (Carl Zeiss Microscopy GmbH, Jena, Germany) was used for analyzing the surface of the samples after sputtering the surface (Au/Pd) with a sputter coater (SCD 500 Bal-Tec, Baltic Präparation e.K., Niesgrau, Germany). Cross sections of the membranes were obtained by using an ion beam milling system (Leica EM TIC 3X, Wetzlar, Germany). The membranes, after the cell measurements, were handled as follows: After the cell tests the pouch-bag cells were opened and the separator membranes were put in dimethyl carbonate for 10 min under shaking conditions. Thereafter, the membranes were washed in 2-propanol. Finally, the membranes were dried at room temperature and at 70 °C (under a vacuum of 1 mbar). 

Air permeability. The air permeability was measured with a Gurley densometer (4110N, rycobel group, Uffenheim, Germany). The measuring surface for the membrane sheets was 1 square-inch. All measurements were performed at room temperature (25 ± 2 °C). 

Drop shape analysis. The contact angles were measured according to the sessile drop method, using an OCA 20, from DataPhysics Instruments GmbH (Filderstadt, Germany). The device was placed in a glovebox, which was perfused with dried compressed air (AD70L from Peak scientific, Inchinnan, Scotland, UK) was used for drying the compressed air). The water content in the box was determined by a dew transmitter (Easidew, Michell Instruments GmbH, Friedrichsdorf, Germany; −100 to 20 °C (±2 °C)) from Michell Instruments, to be less than −58 ± 2 °C. The membranes were fixed with tape (3M Deutschland GmbH, Neuss, Germany, 50 µm; temperature stable up to 150 °C) onto a glass surface for the measurements, dried at 50 °C for 24 h in a vacuum and stored afterwards in a closed glass jar inside the glovebox. The electrolyte drops were supplied by dosage needles (Nordson Deutschland GmbH, Erkrath, Germany; 0.41 mm, 1.5‘‘, 7018266). The electrolyte was composed of ethylene carbonate, dimethyl carbonate (50/50 wt %/wt %) and 1 M LiPF_6_, which is a typical electrolyte composition for lithium ion batteries. The temperature was detected to be 25–27 °C. At least 10 drops, at different positions on the material, were analyzed. A video file of each drop was recorded to detect the contact angles immediately after dropping and over time. The final value was determined by calculating the mean and the standard error (SE). All contact angles given in the manuscript are provided as value ± SE. The software, SCA20 (v 3.7.4 build98), from DataPhysics (DataPhysics Instruments GmbH, Filderstadt, Germany) was used for the data processing of the resulting video file. 

Specific surface. The specific surface was measured by the device, Gemini VII 2390a Surface Analyzer, from Micromeritics (Micromeritics Instrument Corporation, Norcross, Georgia, USA). The membranes were dried at 65 °C in a vacuum; nitrogen adsorption isotherms were measured and monomolecular capacities were calculated. Based on these results, specific surface values were derived from the monomolecular capacity based on the mean of the area of an absorbed nitrogen molecule in the monomolecular layer.

Solvent uptake and porosity. The solvent uptake (*η*) was studied for *n*-decane. Dried membrane sheets of exactly 4.0 × 4.0 cm^2^ were cut out and weighted (*W*_0_). Then, these pieces were placed into the solvent for 48 h. Thereafter, the solvent-soaked sheets were surface-dabbed and the weight was determined (*W*_1_). The solvent uptake (*η*) was calculated according to Equation (2). The porosity (*P*) was determined in a liquid absorption test, by applying Equation (3) [26,27]. The volume (*V*_0_) of the separator was calculated based on its dimensions, *A* (area) and *d* (thickness), where *d* was determined by the medium value from five positions within the 4 × 4 cm^2^ sheet. Additionally, the density of *n*-butanole (*ρ*_sol_ = 0.81 g∙cm^−3^) or *n*-decane (*ρ*_sol_ = 0.73 g∙cm^−3^) was used.

(2) $$\eta =\;\frac{W_{1}-\;W_{0}}{W_{0}}\cdot 100\%$$

(3) $$P=\;\frac{W_{1}-W_{0}}{\rho_{sol}\cdot V_{0}}$$

Tortuosity measurements. The tortuosity (*τ*) can be calculated according to Equation (4), where *P* is the porosity, *κ*_e_ is the conductivity of the electrolyte solvent and *κ*_s_ is the conductivity of the electrolyte-soaked separator membrane [28].

(4) $$\tau =\;\sqrt{\frac{P\cdot \kappa_{e}}{\kappa_{s}}}$$

Conductivity Measurements. The ionic conductivity of the membranes soaked with electrolyte composed of ethylene carbonate and dimethyl carbonate (50:50 wt %) + 1 M LiPF_6_ was measured by a standard complex impedance method, using a Zahner Zennium IM6 electrochemical workstation (Zahner-elektrik I. Zahner-Schiller GmbH & Co. KG, Kronach-Gundelsdorf, Germany), in the frequency range of 1 kHz to 1 MHz. The wetted membrane was placed between two blocking electrodes (stainless steel) in a Swagelok type cell configuration. At the phase minimum of 0°, the impedance value |$\vec{Z}$| was used as the ohmic resistance (*R*) for calculating the specific conductivity (*κ*), according to Equation (5).

(5) $$\kappa =\;\frac{d}{A}\cdot \frac{1}{R}$$

Tensile Testing. The load carrying capacity of the membranes was tested based on the EN ISO 527-3, using a universal testing machine (Zwick GmbH & Co. KG, Ulm, Germany) with a 2.5 kN load cell (accuracy class 0.5). The crosshead speed was 5 mm/min and the stiffness of the testing setup was corrected using a copper dummy sample, to account for the differences in sample strain and cross head displacement. The samples were prepared as coupons, with a width of 15 mm and a gauge length, in between the clamps, of 50 mm. As results from the load–displacement curves—the Young’s modulus (*E*_mod_), yield strength (*σ*_y_), tensile strength (*σ*_max_) and maximum strain (*ε*_max_)—were evaluated, they provided characteristic values for the mechanical performance of the material in the later application as a component.

## 3. Results and Discussion

In this study, the polymer PVdF-HFP was investigated according to its solubility, based on the Hansen approach. On the basis of these findings, micron-sized pored membranes can be prepared from suitable solvent mixtures. The preparation of the membranes was done in a straightforward manner; however, the membrane formation process depends on various influencing factors, like temperature, gelation, reaction time, solvent ratios, extraction time and so on, which have to be controlled strictly to allow reasonable reproduction. Obviously, the porous membrane cannot be heated above its melting point, which is ~135 °C in case of PVdF-HFP. 

### 3.1. Hansen Solubility

Within the Hansen approach, the solubility of a polymer matrix in different solvents can be figured out. In the first step, various solvents were tested in terms of polymer solubility (Table S1, see Supplementary Materials). Based on these experimental data, Hansen spheres of the polymer, PVdF-HFP were calculated. Solvents or solvent mixtures, which are inside these spheres, are most likely able to solve the polymer. These solvents are called “good” solvents or *solvents*. Other organic solvents, in which the polymer is insoluble, are called “bad” solvents or *non-solvents*. 

Based on the co-polymeric structure of PVdF-HFP, two spheres were assumed in the Hansen space for the polymer. With HSPiP software (5th Ed., 2015, Steven Abbott TCNF Ltd, Ipswich, UK) it was possible to use this double sphere fitting procedure, based on experimental solubility tests of solvents and non-solvents [29]. However, it was not possible to achieve any convergent fitting results in the software module with the dataset. The best fit was received with a fit-level of 0.972 (ideal level = 1.000), resulting in two spheres (*δ*_D_, *δ*_P_, *δ*_H_; *R*_0_) for PVdF-HFP (Table 1, Figure S1 in Supplementary Materials). In this case, two organic solvents, out of the 71 tested organic solvents, were wrongly-matched (solvent outside of the sphere or non-solvent inside of the sphere). It can be seen from Table 1 that within the software routine, it is possible neither to determine a true value nor to calculate an average value. The assumption of a double sphere characteristic for the PVdF-HFP polymer was verified by fitting the data with only one sphere. It was proven (Table S2, see Supplementary Materials) that the fitting accuracy of one sphere was significantly inferior than a double sphere fitting. 

A systematic fitting procedure for two spheres was developed in order to simplify the fitting process as following: On the first step, the center (*C*) of all solvents was calculated in the Hansen space. Subsequently, the solvent in the Hansen space, with the maximal distance from *C* was determined (called *M*) and each distance *D* between all solvents and *M* (*D*–*M*) as well as all solvents and *C* (*D*–*C*) was calculated. All solvents with a distance of *D*–*M* < *D*–*C* were collected into one sphere and the center (three-dimensional center) of this sphere was calculated (sphere 1). The radius of this sphere (*R*_0_) was restricted by the closest distance to a non-solvent around the sphere. The other solvents were collected into sphere 2; sphere 2 was calculated accordingly, with all other solvents. Likewise, the closest non-solvent restricted the radius of sphere 2. Finally, two spheres were calculated for the PVdF-HFP polymer (*δ*_D_, *δ*_P_, *δ*_H_; *R*_0_) and are listed in Table 1 as follows: sphere 1 (15.9, 7.7, 7.3, 4.8) and sphere 2 (17.2, 14.6, 7.1, 4.4). Based on the stringent double-sphere protocol, repeated fitting procedures always resulted in the same coordinates. In this case, four of the 34 solvents were outside of the two spheres. The diagrams are depicted in Figures S2 and S3 in the Supplementary Materials. 

**Figure 1:**
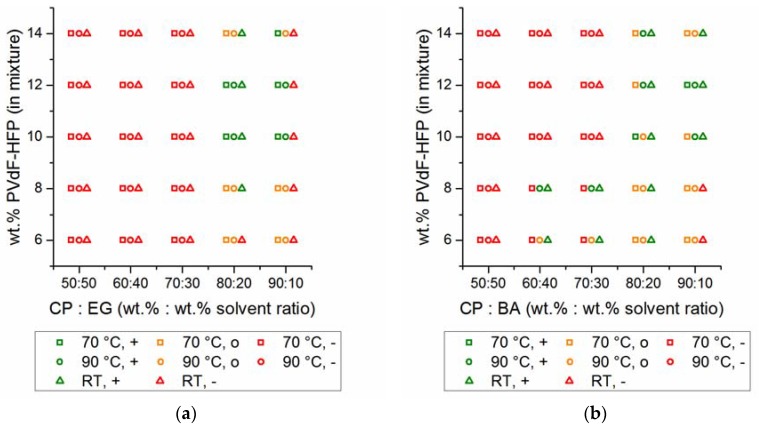
Selected mixtures of (**a**) CP:EG and (**b**) CP:BA, including polyvinylidene difluoride hexafluoropropylene (PVdF-HFP). Symbols are used as follows: square = 70 °C, circle = 90 °C, triangle = room temperature (RT, 25 °C) after cooling from 90 °C. The processability at high temperatures (70 °C/90 °C) is shown in green (appropriate viscosity/homogeneity; 0.5 Pa∙s < *η* < 3.5 Pa∙s at a shear rate of 100 s^−1^), in orange (too high or too low viscosity for processability, which means: *η* > 3.5 Pa∙s at a shear rate of 100 s^−1^ or *η* < 0.5 Pa∙s at a shear rate of 100 s^−1^) or red color (polymer is not dissolved completely, no homogeneous mixture). The consistency at room temperature (after cooling from 90 °C) is shown in green (homogeneous solid) or red (inhomogeneous, not solid, polymer partial precipitated).

After the solubility tests for calculating the Hansen spheres, appropriate solvents and non-solvents were selected based on their physico-chemical properties, toxicity and safety, namely the solvents: cyclohexanone, cyclopentanone (CP), dimethyl sulfoxide and triethyl phosphate and the non-solvents: ethylene glycole (EG), benzyl alcohol (BA) and anisole (for safety and chemical properties see Supplementary Materials, Table S3). Mixtures thereof were analyzed afterwards, according to the relative energy difference (RED) between solvents and polymers, as a pre-screening (see Supplementary Materials, Section 6). In the next step, selected promising solvent/non-solvent blends were investigated experimentally, in regards to their solubility power, in terms of PVdF-HFP. Processability and handling were also analyzed, in addition to solubility characteristics. As a result, two systems were identified for further studies at an ambient PVdF-HFP concentration of ~10 wt %, namely CP:EG (M-1) and CP:BA (M-2). In Figure 1, the processability of both mixtures is shown at selected solvent ratios and PVdF-HFP concentrations. It can be seen that for 80:20 solvent ratios (solvent:non-solvent wt %:wt % ratio) and at PVdF-HFP concentrations of 10–12 wt %, the best processability for these mixtures is achieved. These mixtures can be handled at higher temperatures (70 °C and/or 90 °C) with sufficient viscosity occurring in the range between *η* = 0.5–3.5 Pa∙s (at a shear rate of 100 s^−1^) and result in solid, as well as homogeneous, films at room temperature. 

### 3.2. Preparation via Phase Inversion Techniques

**Figure 2:**
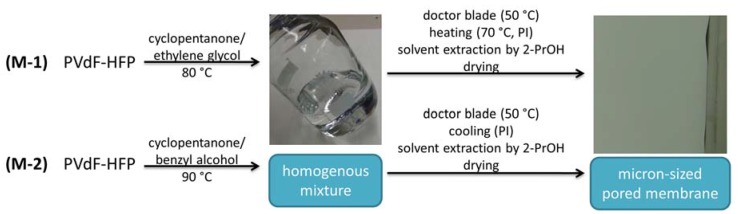
Preparation processes for both micron-sized pored membranes via phase inversion.

The micron-sized pored membranes were prepared by two different approaches, as outlined in Figure 2. The phase inversion process in the first process (M-1) was achieved by evaporating cyclopentanone in a heating step. During this process, the solubility limit of PVdF-HFP in the CP/EG mixture was exceeded and a phase separation took place. After solvent extraction (i-PrOH) and drying, a white non-transparent foil was received. Based on the evaporation process, two different surfaces of the membranes were obtained. In the second process, PVdF-HFP was dissolved in a mixture of cyclopentanone and benzyl alcohol at an increased temperature (~90 °C). The slightly higher temperature was chosen based on the relatively high solidification temperature of 40–50 °C. By cooling off after applying the mixture onto a substrate, the solubility limit of the polymer was exceeded and a porous membrane was formed. Likewise, the membrane was received after solvent extraction and drying. With this approach, a more uniform structure was received, with comparable surfaces on both sites. In this context it is important to mention that the structure of the membrane depends on a variety of individual steps. Thus, the control of individual steps needs particular attention during the preparation process, which limits large-scale manufacturing of these membranes. 

### 3.3. SEM Analysis

**Figure 3:**
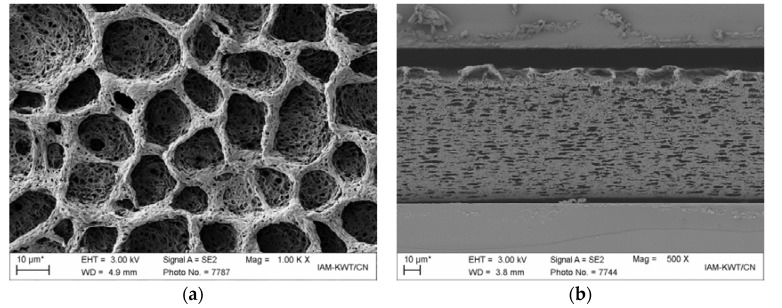

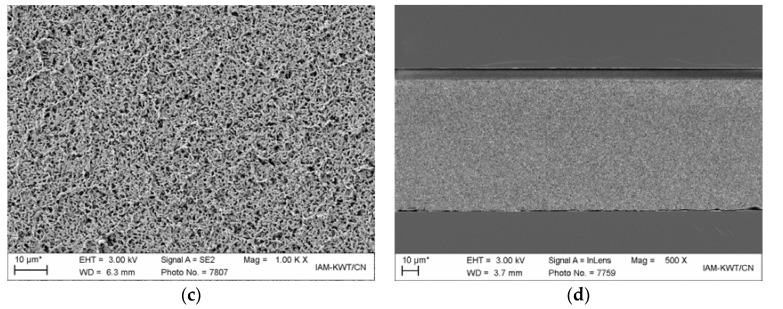
SEM images of the surface of membrane M-1 (**a**,**b**) and M-2 (**c**,**d**) in selected magnifications. (**a**,**c**): top side after membrane preparation; (**b**,**d**): cross section of the membrane. See Figures S4 and S5 (see Supplementary Materials) for detailed SEM images.

Surfaces and cross sections of both membranes were investigated by SEM analysis, which is shown in Figure 3a,b (membrane M-1) and Figure 3c,d (membrane M-2) in selected magnifications. All pictures are representatives which are comparable in various quarters of the membrane layer. Membrane M-1 exhibited two entirely different micro-surface structures (see Supplementary Materials, Figure S4), namely large pores on top of the surface and a homogenous micron-sized pored structure on the bottom side (Figure 3b). The phase inversion process was induced by partial removal of cyclopentanone, which was evaporated through the upper side. Thus, the large pores (d ~ 10–20 µm) arose from the formation of solvent bubbles. The fine porous structure, in terms of shape and pore size, was similar to the bottom side. Within the cross section, no homogenous layer is observed (Figure 3b) and the pore size was inhomogeneous. Nevertheless, the pore distribution in the interior of the membrane was rather uniform. Relatively dense areas (pore diameter of <750 nm) and areas with large pore structures (pore size of 5–18 µm) coexisted simultaneously. In contrast, the cross section of membrane M-2 revealed an almost uniform pore structure (Figure 3d). In this case, phase inversion was induced by a temperature decrease, which was able to form a homogenous structure with no material flow through the membrane. It can be seen that the bottom side of membrane M-2 was less porous than the upper side (Figure 3c). It is assumed that the contact with the stainless steel plate at the bottom side caused this denser structure, due to the slower process of phase separation (slower process of pore structure forming due to slower cooling rate). Presumably, the main differences between both membranes arose from the different mechanisms of the membrane formation, due to solvent evaporation and, solvent extraction. 

In the last step of the preparation process, iso-propanol was removed from the membrane. This was done by placing the membrane onto a stainless steel plate and heating the membrane gently (40–60 °C). It is possible to put the membrane onto a perforated filter (stainless steel) to improve the removability of the dry membrane from the substrate and to enable the removal of solvents from both sites (to improve the surface porosity). It should be noted that the adherence of the membrane to the surface during i-PrOH removal, hampers the shrinkage of the membrane in its dimensions, which usually occurs due to placement of the i-PrOH-soaked membrane onto a paper towel. Detailed SEM images are depicted in the Supplementary Materials (Figures S4 and S5).

### 3.4. Membrane Characteristics: Membrane Characteristics and Mechanical Strength

Both membranes were investigated in regards to their main properties and their mechanical strengths. The parameters of the individual measurements are summarized in Table 2. 

Air permeability values, in the order of 400 Gurley (M-1) or 600 Gurley (M-2), were received for both membranes, which was in the order of tri-layered PP/PE/PP separators [8]. This result states that the air penetrability is more hindered in the dry membrane M-2 than in membrane M-1, which can be ascribed to a denser bottom layer and the smaller porous structure (Figure 3b vs. Figure 3d). The solvent uptake of the membranes was measured by soaking *n*-decane, based on non-solubility and swelling characteristics. It can be seen that the solvent uptake of membrane M-1 was more pronounced, thus the porosity P of membrane M-1 was significantly higher. It should be noted that the porosity value is dependent, more precisely, on the solvent and the swelling of the membrane in the solvent. Usually, *n*-butanol is used for determining the porosity values. For the membranes studied in this manuscript, it was observed that the swelling in *n*-butanol was more pronounced; therefore, *n*-decane was chosen instead of *n*-butanol. The larger porosity value (larger cavity space) of membrane M-1 can be ascribed to much larger pore sizes in membrane M-1. In contrast, the cavity surface of membrane M-1 was lower than in membrane M-2, as evidenced by the BET surface measurements which exhibit values of 5.73 m^2^·g^−1^ (M-1) and 15.80 m^2^·g^−1^ (M-2), respectively. Thus, the number of pores in membrane M-2 is much higher whilst the pore size is smaller than that of M-1. Specifically, a rough calculation for an estimation including the assumption of (a) spherical pores and (b) porosity, membrane size and surface area values, as listed in Table 1, results in pore size numbers of 9.8 × 10^11^ (M-1) vs. 4.6 × 10^13^ (M-2) and pore size diameters of 0.7 µm (M-1) vs. 0.2 µm (M-2). The pore size distribution was inhomogeneous for both membranes. 

**Figure 4:**
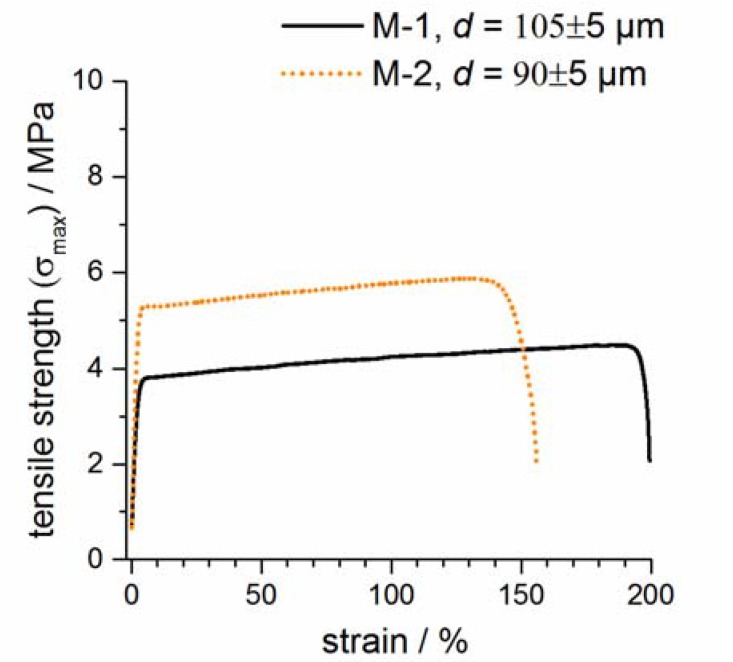
Representative tensile tests of both membranes are shown. The thickness (*d*) of both membranes are *d* (M-1) = 107 ± 10 µm and *d* (M-2) = 91 ± 9 µm (standard deviation).

The mechanical strengths of both dry membranes were tested within tensile tests. Representatives of both membranes are depicted in Figure 4. It can be observed that membrane M-2 had a significantly higher elastic range compared to membrane M-1 (factor of 1.4). This can be attributed to the more homogeneous pore-structure of M-2. In addition, the large pores on the surface as well as in the interior of membrane M-1 weaken the membrane structure significantly. On the other hand, the malleable deformation of M-1 before fracture is larger (M-2 was slightly thinner in the test than membrane M-1). 

### 3.5. Membrane Characteristics: Membrane–Solvent Interactions

Both membranes are investigated regarding their solvent–membrane interactions. All individual parameters of the measurements are summarized in Table 3. 

In potential applications (e.g., lithium ion batteries), the membranes should not limit electrical performance. Nevertheless, the presence of a separator increases the effective resistivity of the electrolyte by a factor of 2–4. The ratio of the resistivity of the separator filled with electrolyte divided by the resistivity of the electrolyte alone is called the McMullin number. McMullin numbers as high as 10–12 have been used in Li-ion battery cells. Ionic conductivity was measured from electrolyte soaked (24 h) membranes. Surprisingly, in spite of the large pore structures (SEM), the denser bottom surface (SEM) and the higher porosity (Table 1) of membrane M-1, significantly higher conductivity values were received for membrane M-2 than for membrane M-1 (factor of ~2). These conductivity values can be reproduced at different membrane positions. Contrary to the assumption based on the porosity and pore size of the dry membrane, the tortuosity of membrane M-2 was significantly lower than in the case of membrane M-1. Based on the membrane swelling in the electrolyte solvent, the dense bottom structure of membrane M-2 is assumed to become more permeable than the SEM picture of the dry membrane suggests. Thus, the high pore number and the higher conductivity value of membrane M-2 account for the reduced tortuosity value.

The contact angle between the solvent and the micron-sized pored membrane was studied with an EC/DMC + 1 M LiPF_6_ based electrolyte and carefully dried separator sheets. Immediately after taking the drop, the contact angle was highly dependent on the surface side in the case of membrane M-1, whereas no distinct difference in contact angle could be observed for both surface sides for membrane M-2. The behavior of membrane M-1 is due to the significantly different surface fine structures of both surface sides for membrane M-1 (Figure 3a,c). The infiltration times clearly differed between both membranes. The times that were necessary for the drop to form a contact angle of 20° between the drop and the membrane surface were quite different. Almost immediate infiltration was observed for the top side of membrane M-1 (Figure 3a). Slightly slower solvent uptake was detected for the downside of membrane M-1, and a denser surface structure reduced the infiltration time. However, a much slower solvent uptake was observed for membrane M-2, due to significantly smaller pore sizes and an increased polymer surface. Thus, the polar solvent could not infiltrate easily into the interior of the membrane, but needed longer time or supporting techniques (e.g., applying a vacuum) [30]. 

## 4. Conclusions

In this manuscript, two processes of micron-sized pored membrane preparation based on the co-polymer PVdF-HFP are presented. The membranes were fabricated by two selected phase inversion methods, with non-toxic and low-flammable solvent formulations. Up until now, such membranes were prepared with flammable acetone/PVdF-HFP or toxic solvents (methanol, dimethyl formamide, *N*-methyl pyrrolidone). Thus, the novel approach can be used as safer and/or more green preparation method for micron-sized pored PVdF-HFP membranes. Appropriate solvent mixtures were selected through application of the Hansen solubility method. The Hansen parameters of the polymer were determined experimentally with a setup of 71 chemicals. Ionic conductivities of both membranes were measured with standard electrolyte formulation solvents and McMullin numbers of 3.8 and 1.9 were calculated, respectively. Additionally, mechanical properties and the interaction of membrane and selected solvents were quantified. The approach can be used for the preparation of other pored polymer membranes with similar phase inversion techniques.

## Figures and Tables

**Table 1:** Comparison of software fitting and proposed fitting method.

Fitting Procedure ^1^	Fit Accuracy	Wrong Correlated Solvents (s) and Non-Solvents (ns) ^2^	Sphere 1				Sphere 2			
			*δ*_D_	*δ*_P_	*δ*_H_	*R*_0_	*δ*_D_	*δ*_P_	*δ*_H_	*R*_0_
A-1	0.972	1 s out, 1 ns in	15.7	8.8	7.1	5.6	17.9	15.8	8.3	6.5
A-2	0.972	1 s out, 1 ns in	18.1	16.1	9.2	6.9	14.9	9.1	7.1	6.2
A-3	0.972	1 s out, 1 ns in	15.4	0.6	8.2	4.5	15.4	13.2	8.4	8.5
B	---	4 s out	15.9	7.7	7.3	4.8	17.2	14.6	7.1	4.4

^1^ Method A = Fitting procedure according to HSPiP software; Method B = Fitting procedure according to the method described in the manuscript text. ^2^ Wrongly-correlated solvents = solvents which are outside of both spheres (called “out”); wrongly-correlated non-solvents = non-solvents which are located in one or both spheres (called “in”).

**Table 2:** Membrane characteristics and mechanical stabilities of both membranes. Provided are the mean value and the standard error (SE) of the mean value from *n* individual measurements.

Parameter	M-1	M-2
Gurley related to a membrane thickness of 25 µm (/100 cm^3^) (*n* = 5) according to (permeability∙25)/(membrane thickness [in µm])	114 ± 7	131 ± 26
typical membrane thickness (µm)	80–110	80–120
typical membrane weight (mg/cm^2^) (*n* = 6)	95 ± 2	93 ± 2
solvent uptake (*n*-decane) related to membrane thickness of 25 µm (µL/cm^2^) (*n* = 3)	1.74 ± 0.09	1.51 ± 0.03
solvent uptake (*n*-decane) (% related to membrane weight) (*n* = 3)	88 ± 6	69 ± 2
porosity (*P*) (*n* = 6) based on solvent uptake	70 ± 2	56 ± 2
pore size distribution (SEM)	inhomogeneous	inhomogeneous
pore size (maximum length; SEM)	<18 µm	<1.4 µm
Brunauer-Emmett-Teller (BET) surface area (m^2^/g)	5.73	15.80
*E*_mod_/GPa (*n* = 3)	0.16 ± 0.06	0.25 ± 0.08
*σ*_y_/MPa (*n* = 3)	3.5 ± 0.1	5.1 ± 0.3
*σ*_max_/MPa (*n* = 3)	4.35 ± 0.09	5.90 ± 0.02
*ε*_max_	1.7 ± 0.2	1.0 ± 0.2

**Table 3:** Characteristics of membrane–solvent-interactions of both micron-sized pored membranes. Provided are the mean value and the standard error (SE) of the mean value from *n* individual measurements.

Parameter	M-1	M-2
McMullin number; electrolyte: EC/DMC^(a)^ 1:1 wt % + 1 M LiPF_6_	3.7	1.9
ionic conductivity (mS·cm^−1^) (*n* = 5)^(b)^	3.2 ± 0.2	6.0 ± 0.2
tortuosity	1.6	1.0
contact angle immediately after take drop (°) (top)	27 ± 2 (*n* = 9)	48 ± 1 (*n* = 37)
contact angle immediately after take drop (°) (bottom)	41 ± 1 (*n* = 9)	49 ± 2 (*n* = 30)
time up to contact angle of 20° (s) (top)	<2 (*n* = 17)	104 ± 6 (*n* = 37)
time up to contact angle of 20° (s) (bottom)	18 ± 2 (*n* = 17)	106 ± 7 (*n* = 30)

^(a)^ The electrolyte solvent is composed of ethylene carbonate (EC) and dimethyl carbonate (DMC). ^(b)^ The electrolyte mixture is composed of EC/DMC (1:1 wt %) + 1 M LiPF_6_. The conductivity of the pure electrolyte is 10.4 mS·cm^−1^ at 25 °C.

## Supplementary Materials

The following are available online at www.mdpi.com/2073-4360/9/10/489/s1; Figure S1: Illustration of the double spheres of PVdF-HFP based on the HSPiP software, Figure S2: Projection of the Hansen sphere in the dD-dP dimension, Figure S3: Projection of the Hansen sphere in the dP-dH dimension, Figure S4: SEM images of membrane M-1, Figure S5: SEM images of membrane M-2. Table S1: Hansen solubility parameter of selected solvents, Table S2: Comparison of software fitting and proposed fitting method, Table S3: Safety properties of chemicals according to safety data sheets taken from Bernd Kraft GmbH, Table S4: Comparison of experimental and calculated solubility. Solubility of PVdF-HFP in selected solvent mixtures based on Hansen calculation. Section 6: Solubility of mixtures between solvents and non-solvents according to Hansen approach.
