# Supplementary material for: Micron-Sized Pored Membranes Based on Polyvinylidene Difluoride Hexafluoropropylene Prepared by Phase Inversion Techniques
Source: Polymers (Basel). 2017 Oct 6;9(10):489. doi: 10.3390/polym9100489 (PMC6418560; doi:10.3390/polym9100489)
Supplement: Supplementary file 1 [file polymers-09-00489-s001.docx]

**Supporting Information**

1. *Hansen solubility parameters*

| Table S1. Hansen solubility parameter of selected solvents [1, 2]. Experimental evaluation of the Hansen solubility of PVdF-HFP. All solvents are used in purity of 98% or higher. The classification “soluble” (1 = yes) means in this context that a *homogeneous* liquid or gel-like behavior was received. Some of the samples which were not or very poor soluble at room temperature were heated up to 50 °C for 24 h. After cooling down to room temperature, these samples were classified again. Samples which remain homogeneous and liquid or gel-like at 20-25 °C were also classified as “soluble”. This classification was used in the HSPiP software as well as in the described fitting protocol. | | | | | |
| --- | --- | --- | --- | --- | --- |
| **dD** | **dP** | **dH** | **soluble**  (0 = no,  1 = yes) | **number for plot** | **solvent** |
| 16.6 | 8.1 | 20.90 | 0 | M1 | 1,3-Butanediol |
| 17.5 | 1.8 | 9.0 | 1 | N1 | 1,4-Dioxane |
| 16.0 | 5.7 | 15.8 | 0 | O1 | 1-Butanol |
| 16.0 | 5.0 | 11.2 | 0 | P1 | 1-Octanol |
| 15.8 | 5.7 | 14.5 | 0 | Q1 | 2-Butanol |
| 17.3 | 2.8 | 7.4 | 0 | U1 | 2-Methylfuran |
| 16.2 | 12.1 | 4.1 | 0 | R1 | 2-Nitropropane |
| 15.8 | 6.1 | 16.4 | 0 | A | 2-Propanol |
| 15.5 | 10.4 | 7.0 | 1 | C | Acetone |
| 15.3 | 18.0 | 6.1 | 1 | D | Acetonitrile |
| 18.8 | 9.0 | 4.0 | 0 | T1 | Acetophenone |
| 17.8 | 4.4 | 6.9 | 0 | V1 | Anisole |
| 19.4 | 7.4 | 5.3 | 0 | W1 | Benzaldehyde |
| 18.8 | 12 | 3.3 | 0 | E | Benzonitrile |
| 18.4 | 6.3 | 13.7 | 0 | X1 | Benzyl Alcohol |
| 15.3 | 12.4 | 5.1 | 1 | Z1 | Butyronitrile |
| 19.0 | 4.3 | 2.0 | 0 | A2 | Chlorobenzene |
| 17.8 | 3.1 | 5.7 | 0 | F | Chloroform |
| 16.8 | 0.0 | 0.2 | 0 | G | Cyclohexane |
| 17.8 | 8.4 | 5.1 | 1 | B2 | Cyclohexanone |
| 17.9 | 11.9 | 5.2 | 1 | H | Cyclopentanone |
| 19.6 | 3.4 | 5.2 | 0 | C2 | Dibenzyl Ether |
| 15.1 | 6.3 | 3.5 | 1 | I | Diethyl Carbonate |
| 15.8 | 7.6 | 4.7 | 1 | E2 | Diethyl Ketone |
| 16.6 | 12.0 | 19.0 | 0 | D2 | Diethylene Glycol |
| 15.5 | 8.6 | 9.7 | 1 | L | Dimethyl Carbonate |
| 18.4 | 16.4 | 10.2 | 1 | N | Dimethyl Sulfoxide (DMSO) |
| 17.2 | 1.8 | 4.3 | 0 | UW | Dipentene (Dl-Limonene) |
| 15.8 | 8.8 | 19.4 | 0 | O | Ethanol |
| 15.8 | 5.3 | 7.2 | 1 | P | Ethyl Acetate |
| 17.8 | 0.6 | 1.4 | 0 | F2 | Ethyl Benzene |
| 18.4 | 8.2 | 4.1 | 0 | G2 | Ethyl Cinnamate |
| 15.5 | 8.4 | 8.4 | 1 | H2 | Ethyl Formate |
| 16.0 | 7.6 | 12.5 | 1 | I2 | Ethyl Lactate |
| 18 | 21.7 | 5.1 | 0 | Q | Ethylene Carbonate |
| 17.0 | 11.0 | 26.0 | 0 | R | Ethylene Glycol |
| 15.4 | 6.3 | 6.0 | 1 | S | Ethylene Glycol Dimethyl Ether |
| 17.2 | 26.2 | 19.0 | 0 | J2 | Formamide |
| 17.0 | 1.8 | 5.3 | 0 | K2 | Furan |
| 17.4 | 7.6 | 15.1 | 0 | M2 | Furfuryl Alcohol |
| 18 | 16.6 | 7.4 | 1 | T | g-Butyrolactone (GBL) |
| 17.4 | 11.3 | 27.2 | 0 | U | Glycerol |
| 14.9 | 0.0 | 0.0 | 0 | Z | Hexane |
| 18.5 | 6.5 | 13.7 | 0 | W | m-Cresol |
| 14.7 | 12.3 | 22.3 | 0 | X | Methanol |
| 15.5 | 7.2 | 7.6 | 1 | N2 | Methyl Acetate |
| 16.0 | 9.0 | 5.1 | 1 | Y | Methyl Ethyl Ketone (MEK) |
| 18.0 | 4.9 | 11.0 | 0 | O2 | Morpholine |
| 16.4 | 11.4 | 9.2 | 1 | J | N,N-Diethyl Formamide |
| 16.8 | 11.5 | 9.4 | 1 | K | N,N-Dimethyl Acetamide |
| 17.4 | 13.7 | 11.3 | 1 | M | N,N-Dimethyl Formamide (DMF) |
| 15.8 | 3.7 | 6.3 | 1 | Y1 | n-Butyl Acetate |
| 16.0 | 15.5 | 4.5 | 1 | P2 | Nitroethane |
| 15.8 | 18.8 | 6.1 | 1 | Q2 | Nitromethane |
| 18.0 | 12.3 | 7.2 | 1 | A1 | N-Methyl-2-Pyrrolidone (NMP) |
| 15.5 | 0.0 | 0.0 | 0 | B1 | Octane |
| 17.6 | 1.0 | 3.1 | 0 | R2 | o-Xylol |
| 15.3 | 14.3 | 5.5 | 1 | S2 | Propionitrile |
| 20.0 | 18.0 | 4.1 | 1 | C1 | Propylene Carbonate |
| 17.8 | 17.4 | 8.7 | 1 | D1 | Sulfolane (Tetramethylene Sulfone) |
| 16.8 | 5.7 | 8.0 | 1 | G1 | Tetrahydrofuran (THF) |
| 16.7 | 8.2 | 11.0 | 1 | T2 | Tetramethylurea |
| 18.0 | 1.4 | 2.0 | 0 | H1 | Toluene |
| 17.3 | 7.6 | 21 | 0 | I1 | Triethanolamine |
| 16.0 | 12.5 | 18.6 | 0 | E1 | Triethylene Glycol |
| 16.7 | 11.4 | 9.2 | 1 | F1 | Triethylphosphate |
| 15.7 | 10.5 | 10.2 | 1 | V2 | Trimethylphosphate |
| 16.3 | 6.3 | 4.3 | 1 | U2 | Tri-n-Butyl Phosphate |
| 15.3 | 11.0 | 4.8 | 1 | J1 | Valeronitrile |
| 16.0 | 7.2 | 5.9 | 1 | K1 | Vinyl Acetate |
| 15.5 | 16.0 | 42.3 | 0 | L1 | Water |

2. *Hansen spheres*


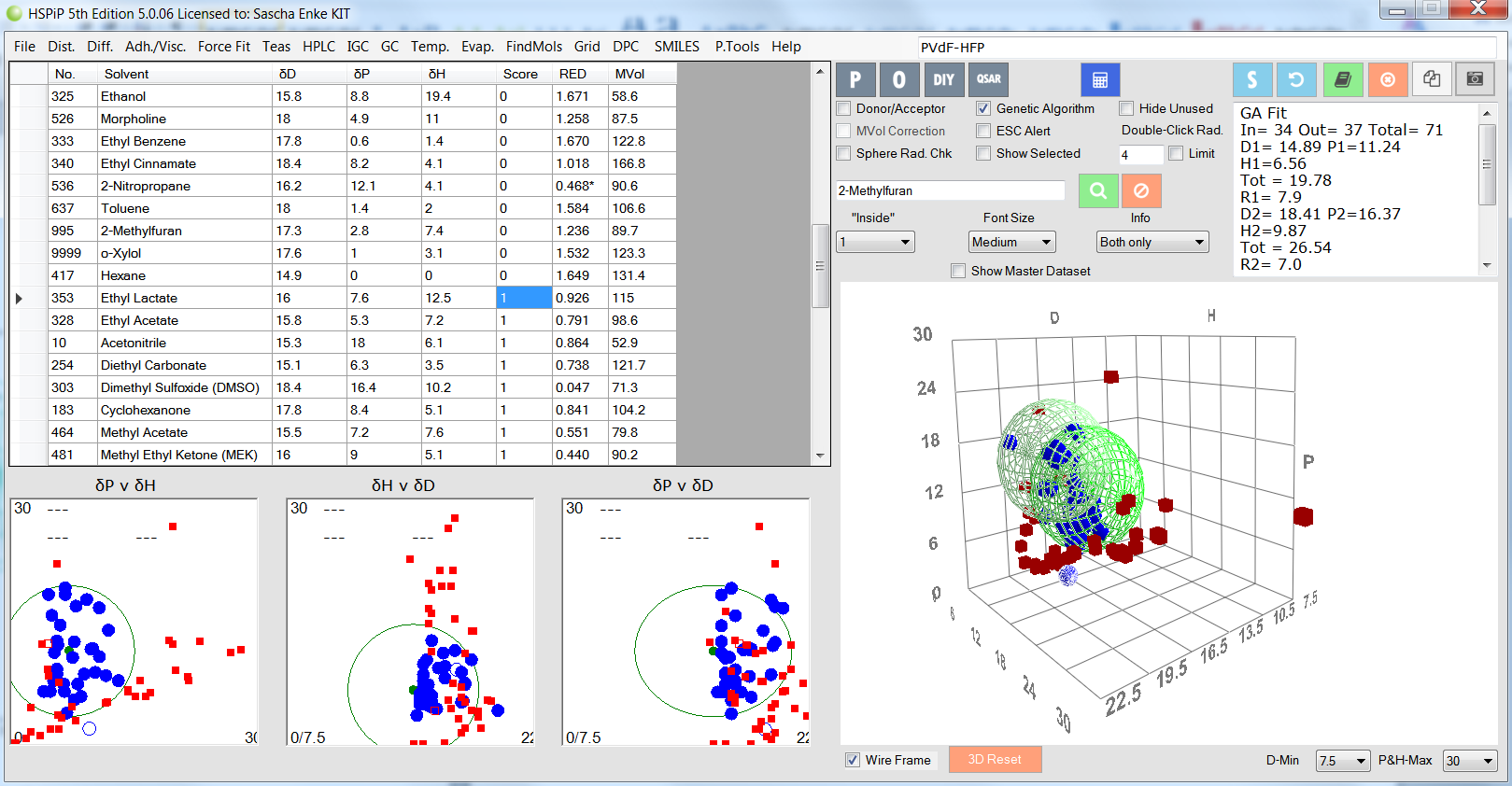


Figure S1. Illustration of the double spheres of PVdF-HFP (#11 of Table 1) based on the HSPiP software (δ*D*, δ*P*, δ*H*; *R*_0_): sphere 1 (14.9, 11.2, 6.6, 7.9) and sphere 2 (18.4, 16.4, 9.9, 7.0).


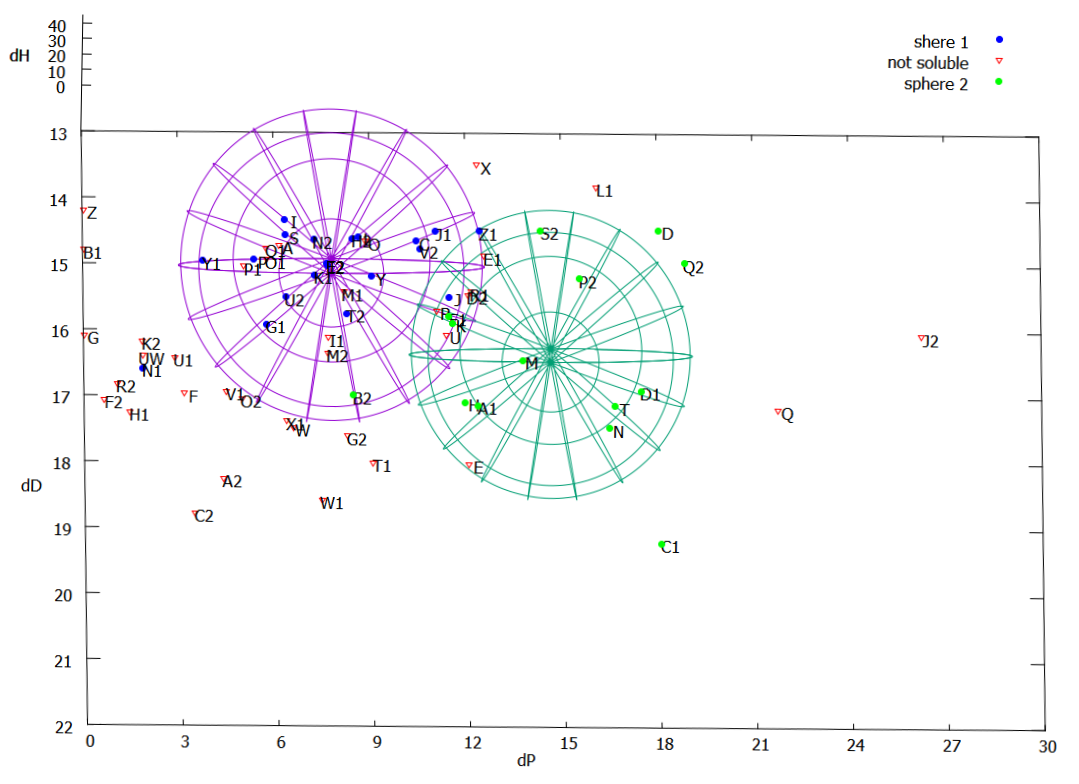


Figure S2. Projection of the Hansen sphere in the dD-dP dimension (calculated as described in the manuscript text).


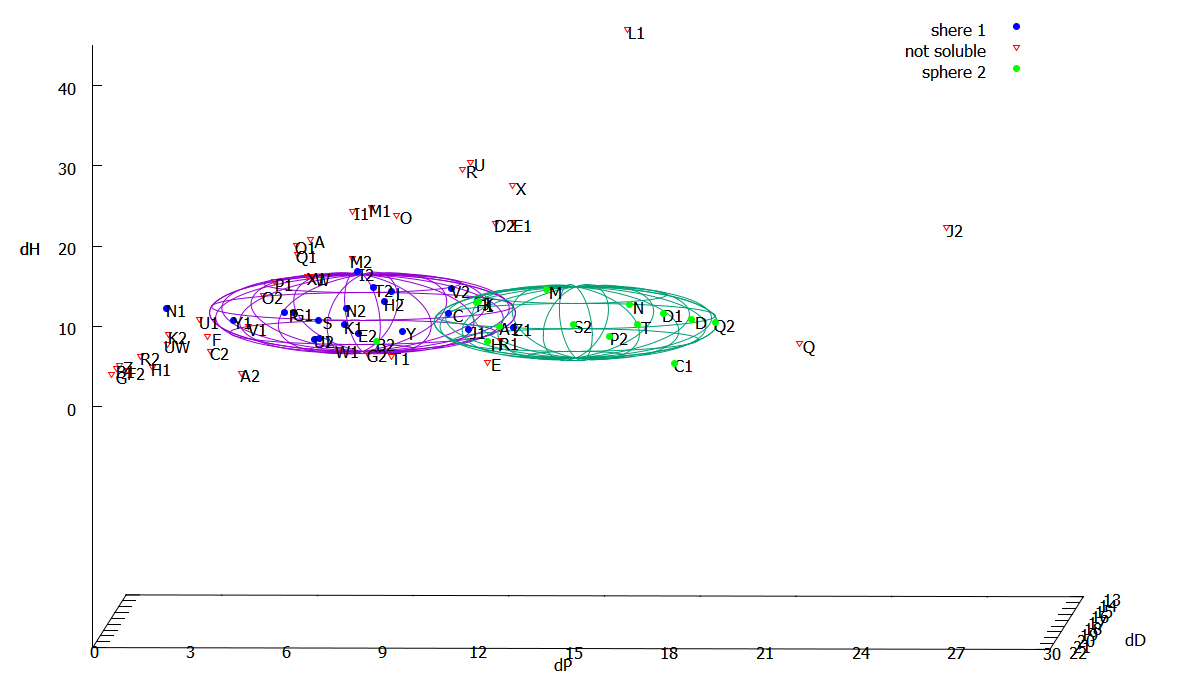


Figure S3. Projection of the Hansen sphere in the dP-dH dimension (calculated as described in the manuscript text).

3. *Hansen fitting methods*

| Table S2. Comparison of software fitting and proposed fitting method. Wrong correlated *solvents*: *solvents* which are outside of both spheres (out); wrong correlated *non-solvents*: *non-solvents* which are located in one or both spheres (in). | | | | | | | | | | | | | | | |
| --- | --- | --- | --- | --- | --- | --- | --- | --- | --- | --- | --- | --- | --- | --- | --- |
| Fitting procedure according to HSPiP software with one sphere (normal fit); 10 independent fitting tests are shown. | | | | | | | | | | | | | | | |
| fit nr. | | Fit accuracy | | wrong correlated solvents (S) and non-solvents (NS) | sphere 1 | | | | | |  | | | | |
|  |  |  |  |  | δ*D* | δ*P* | | δ*H* | | *R*_0_ |  | | | | |
| 1 | | 0.746 | | 5 S out, 5 NS in | 16.1 | 11.5 | | 9.0 | | 7.8 |  | | | | |
| 2 | | 0.746 | | 3 S out, 4 NS in | 15.9 | 11.6 | | 9.1 | | 7.8 |  | | | | |
| 3 | | 0.745 | | 4 S out, 4 NS in | 15.9 | 11.5 | | 9.1 | | 7.8 |  | | | | |
| 4 | | 0.746 | | 3 S out, 4 NS in | 16.0 | 11.6 | | 9.0 | | 7.8 |  | | | | |
| 5 | | 0.737 | | 2 S out, 3 NS in | 13.7 | 14.4 | | 8.0 | | 11.4 |  | | | | |
| 6 | | 0.746 | | 5 S out, 5 NS in | 16.1 | 11.5 | | 9.0 | | 7.8 |  | | | | |
| 7 | | 0.746 | | 5 S out, 5 NS in | 16.1 | 11.5 | | 9.0 | | 7.8 |  | | | | |
| 8 | | 0.745 | | 4 S out, 5 NS in | 16.1 | 11.5 | | 9.0 | | 7.8 |  | | | | |
| 9 | | 0.745 | | 4 S out, 5 NS in | 16.1 | 11.5 | | 9.0 | | 7.8 |  | | | | |
| 10 | | 0.746 | | 3 S out, 4 NS in | 15.9 | 11.6 | | 9.0 | | 7.8 |  | | | | |
| Fitting procedure according to HSPiP software with one spheres according to Genetic Algorithm technique fitting. 10 independent fitting tests are shown. | | | | | | | | | | | | | | | |
| fit nr. | | Fit accuracy | | wrong correlated solvents (S) and non-solvents (NS) | sphere 1 | | | | | |  | | | | |
|  |  |  |  |  | δ*D* | δ*P* | | δ*H* | | *R*_0_ |  | | | | |
| 1 | | 0.944 | | 1 S out, 3 NS in | 15.6 | 12.3 | | 7.7 | | 7.4 |  | | | | |
| 2 | | 0.944 | | 1 S out, 3 NS in | 15.8 | 12.0 | | 8.0 | | 7.4 |  | | | | |
| 3 | | 0.944 | | 1 S out, 3 NS in | 15.4 | 12.3 | | 7.8 | | 7.8 |  | | | | |
| 4 | | 0.944 | | 2 S out, 2 NS in | 14.9 | 12.0 | | 8.3 | | 8.9 |  | | | | |
| 5 | | 0.944 | | 1 S out, 3 NS in | 15.3 | 12.4 | | 7.3 | | 7.9 |  | | | | |
| 6 | | 0.958 | | 1 S out, 2 NS in | 13.8 | 13.3 | | 6.6 | | 10.4 |  | | | | |
| 7 | | 0.944 | | 1 S out, 3 NS in | 15.6 | 12.5 | | 7.9 | | 7.8 |  | | | | |
| 8 | | 0.958 | | 1 S out, 2 NS in | 14.1 | 13.1 | | 6.6 | | 10.0 |  | | | | |
| 9 | | 0.958 | | 1 S out, 3 NS in | 13.4 | 13.8 | | 6.1 | | 11.3 |  | | | | |
| 10 | | 0.944 | | 1 S out, 3 NS in | 15.2 | 12.9 | | 8.0 | | 8.1 |  | | | | |
| Fitting procedure according to HSPiP software with double spheres (fitting accuracy = higher; fit number 1-3 are the same fits as in the manuscript text). 10 independent fitting tests are shown. | | | | | | | | | | | | | | | |
| fit nr. | Fit accuracy | | wrong correlated solvents (S) and non-solvents (NS) | | sphere 1 | | | | | | | sphere 2 | | | |
|  |  |  |  |  | δ*D* | δ*P* | δ*H* | | *R*_0_ | | | δ*D* | δ*P* | δ*H* | *R*_0_ |
| 1 | 0.972 | | 1 S out, 1 NS in | | 15.7 | 8.8 | 7.1 | | 5.6 | | | 17.9 | 15.8 | 8.3 | 6.5 |
| 2 | 0.972 | | 1 S out, 1 NS in | | 18.1 | 16.1 | 9.2 | | 6.9 | | | 14.9 | 9.1 | 7.1 | 6.2 |
| 3 | 0.972 | | 1 S out, 1 NS in | | 15.4 | 0.6 | 8.2 | | 4.5 | | | 15.4 | 13.2 | 8.4 | 8.5 |
| 4 | 0.972 | | 1 S out, 1 NS in | | 14.3 | 11.3 | 6.9 | | 8.2 | | | 19 | 16.6 | 7.1 | 4.8 |
| 5 | 0.972 | | 1 S out, 1 NS in | | 15.7 | 9.6 | 7.1 | | 6.2 | | | 17.9 | 15.7 | 10.1 | 7.8 |
| 6 | 0.972 | | 1 S out, 1 NS in | | 15.2 | 8.9 | 7.1 | | 5.9 | | | 18.1 | 16.5 | 9.4 | 6.7 |
| 7 | 0.972 | | 1 S out, 1 NS in | | 15.9 | 7.3 | 6.9 | | 4.8 | | | 17.4 | 14.6 | 11.1 | 7.7 |
| 8 | 0.972 | | 1 S out, 1 NS in | | 17.9 | 16.3 | 7.9 | | 6.1 | | | 16.1 | 9.1 | 7.4 | 5.5 |
| 9 | 0.972 | | 1 S out, 1 NS in | | 18.0 | 17.0 | 6.4 | | 4.8 | | | 15.0 | 10.8 | 7.1 | 7.3 |
| 10 | 0.972 | | 1 S out, 1 NS in | | 18.6 | 15.9 | 8.8 | | 6.8 | | | 14.3 | 10.4 | 6.5 | 7.9 |
| 11 | 0.972 | | 1 S out, 1 NS in | | 14.9 | 11.2 | 6.6 | | 7.9 | | | 18.4 | 16.4 | 9.9 | 7.0 |
| Fitting procedure according to novel method described in the text | | | | | | | | | | | | | | | |
| fit nr. |  | | wrong correlated solvents (S) | | sphere 1 | | | | | | | sphere 2 | | | |
| 1 | --- | | 4 S out | | 15.9 | 7.7 | 7.3 | | 4.8 | | | 17.2 | 14.6 | 7.1 | 4.4 |

4. *Safety properties of selected solvents*

| Table S3. Safety properties of chemicals according to safety data sheets taken from Bernd Kraft GmbH. | | | | | | |
| --- | --- | --- | --- | --- | --- | --- |
| Property | flash point  [°C] | boiling point  [°C] | vapor pressure (25 °C) [hPa] | ignition temperature [°C] | explosion range [1 atm, vol.%]  lower-upper range | Hazard statements |
| acetone^[1]^ | < -20 | 56 | 233 | 465 | 2.6-12.8 | H225 (!)  H319  H336 |
| dimethyl acetamide^[1,2]^ | 70 | 165-166 | 3.3  (20 °C) | 390 | 1.8-11.5 | H360D (!)  H312  H332 |
| dimethyl formamide^[1,2]^ | 57 | 153 | 3.5  (20 °C) | 440 | 2.2-16 | H226  H360D (!)  H312  H332  H319 |
| NMP^[1]^ | 93 | 202 | 0.3 | 245 | 1.3-9.5 | H315  H319  H360D (!)  H335 |
| anisole^[2]^ | 43 | 153-154 | 0.4  (20 °C) | 475 | 0.34-6.3 | H226  H332  H315  H319  H335 |
| benzylalcohol | 101 | 205 | 0.03 | 435 | 1.3-13 | H302  H332 |
| cyclohexanone | 44 | 154 | 24 | 420 | 1.1-9.4 | H226  H318  H302  H312  H332  H315 |
| cyclopentanone | 26 | 131 | 11.4 | 430 | 1.6-10.8 | H226  H315  H319 |
| dimethylsulfoxide | 95 | 189 | 2.5 | 301 | 1.8-63 | --- |
| ethylene glycole | 111 | 197 | 0.08 | 410 | 1.8-12.8 | H302  H373 |
| triethyl phosphate^[2]^ | 115 | 215 | 0.2  (20 °C) | 457 | 1.7-10 | H302 |
| [1] Commonly used solvents for phase separation reactions. The symbol “(!)” accounts for particular safety risks based on the following selection: material: H200-225; health: H300, 301, 304, 310, 311, 330, 331, 340, 341, 350, 351, 360, 361, 370, 372. [2] Data are taken from Alfa Aesar. | | | | | | |

5. *SEM images of microporous membranes*




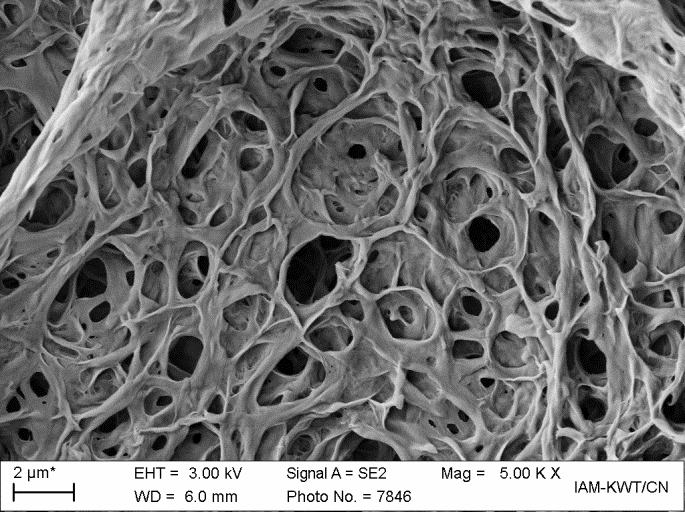


4e

4f

4c

4d

4a

4b


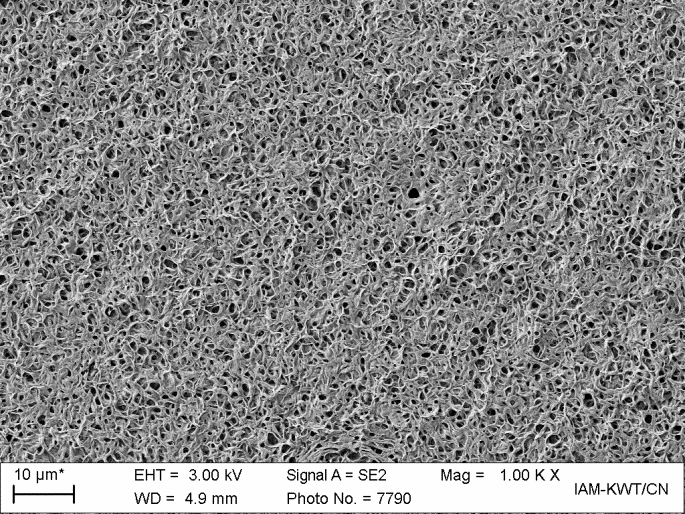

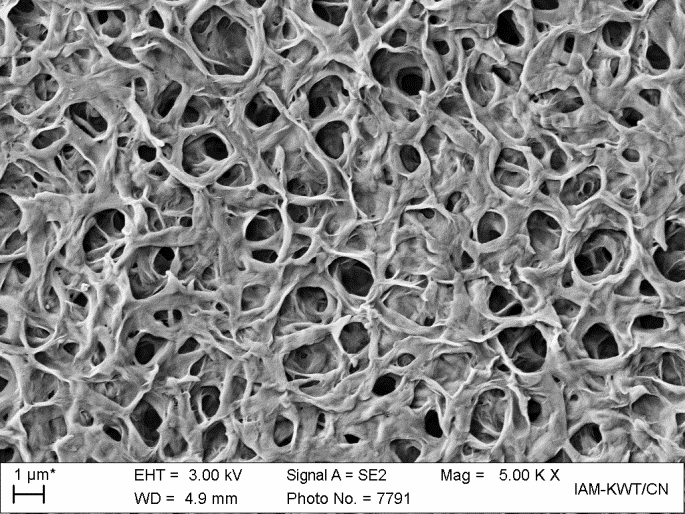




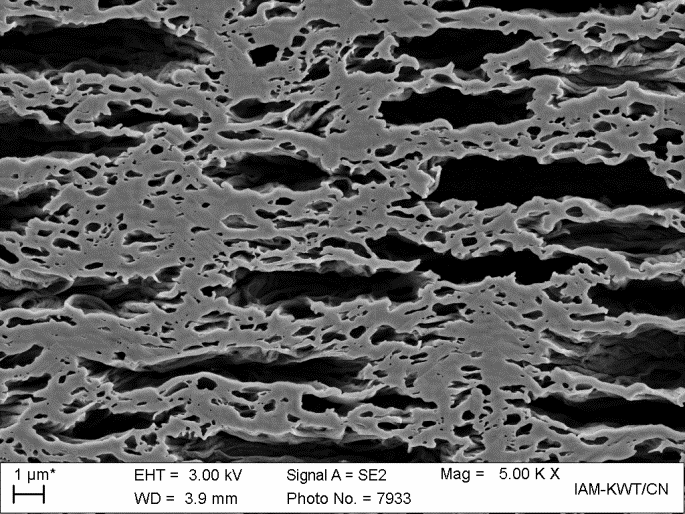


**Figure S4.** SEM images of the surface of M-1 membrane in selected magnifications. 3a/b: top side during PI process; 3c/d: bottom side during PI process; 3e/f: cross section.




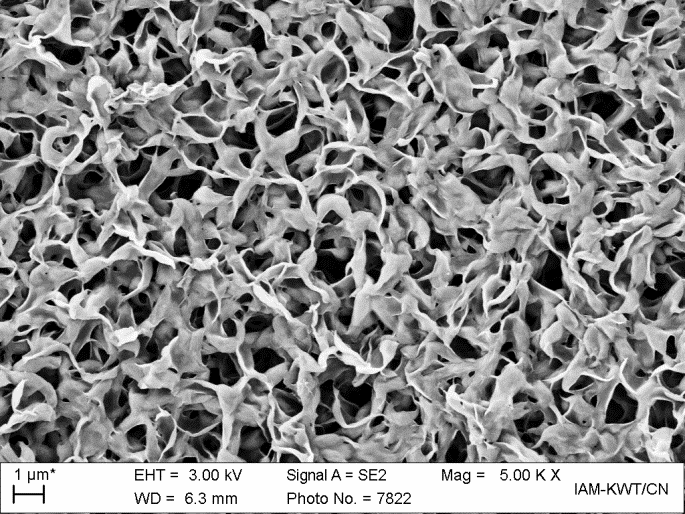


5e

5f

5c

5d

5a

5b


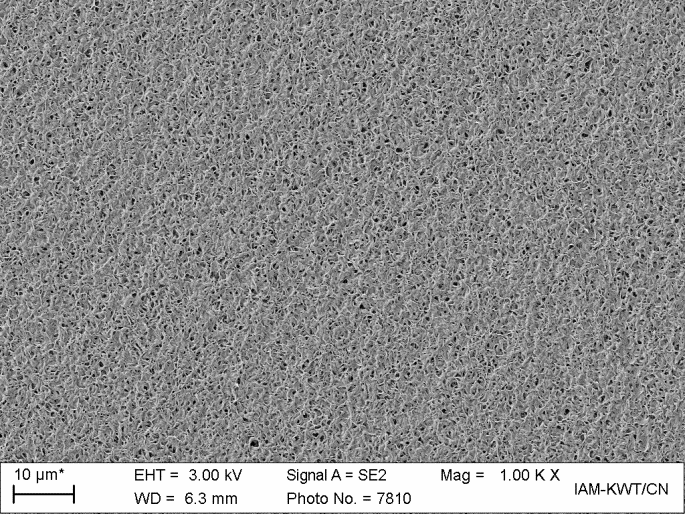

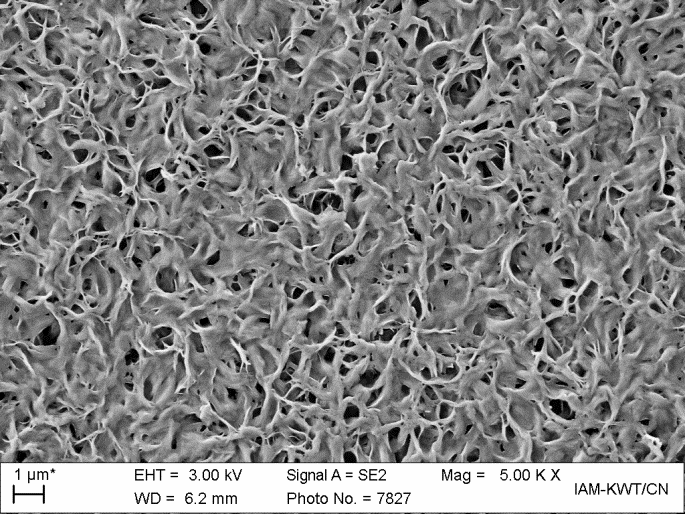




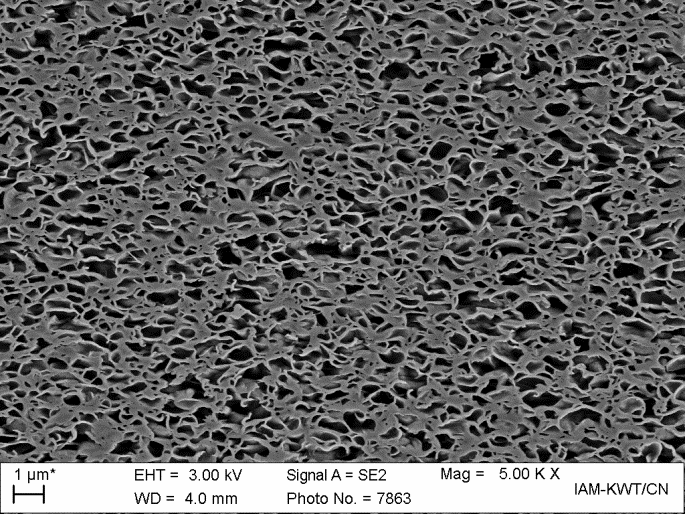


**Figure S5.** SEM images of the surface of M-1 membrane in selected magnifications. 4a/b: topside during PI process; 4c/d: bottom side during PI process; 4e/f: cross section.

6. *Solubility of mixtures between solvents and non-solvents according to Hansen approach [1, 2].*

A) CH + EG

|  | dD | dP | dH | R |  |
| --- | --- | --- | --- | --- | --- |
| Target | 15,9 | 7,7 | 7,3 | 4,8 |  |
| Solvent | dD | dP | dH | Distance | RED |
| Cyclohexanone | 17,8 | 8,4 | 5,1 | 4,45 | 0,93 |
| Ethylene Glycol | 17 | 11 | 26 | 19,12 | 3,98 |
| % S2 |  |  |  |  |  |
| 0 | 17,8 | 8,4 | 5,1 | 4,45 | 0,93 |
| 10 | 17,72 | 8,66 | 7,19 | 3,77 | 0,78 |
| 20 | 17,64 | 8,92 | 9,28 | 4,19 | 0,87 |
| 30 | 17,56 | 9,18 | 11,37 | 5,46 | 1,14 |
| 40 | 17,48 | 9,44 | 13,46 | 7,14 | 1,49 |
| 50 | 17,4 | 9,7 | 15,55 | 9,00 | 1,88 |
| 60 | 17,32 | 9,96 | 17,64 | 10,96 | 2,28 |
| 70 | 17,24 | 10,22 | 19,73 | 12,96 | 2,70 |
| 80 | 17,16 | 10,48 | 21,82 | 15,00 | 3,12 |
| 90 | 17,08 | 10,74 | 23,91 | 17,05 | 3,55 |
| 100 | 17 | 11 | 26 | 19,12 | 3,98 |

|  | dD | dP | dH | R |  |
| --- | --- | --- | --- | --- | --- |
| Target | 17,2 | 14,6 | 7,1 | 4,4 |  |
| Solvent | dD | dP | dH | Distance | RED |
| Cyclohexanone | 17,8 | 8,4 | 5,1 | 6,62 | 1,51 |
| Ethylene Glycol | 17 | 11 | 26 | 19,24 | 4,37 |
| % S2 |  |  |  |  |  |
| 0 | 17,8 | 8,4 | 5,1 | 6,62 | 1,51 |
| 10 | 17,72 | 8,66 | 7,19 | 6,03 | 1,37 |
| 20 | 17,64 | 8,92 | 9,28 | 6,15 | 1,40 |
| 30 | 17,56 | 9,18 | 11,37 | 6,94 | 1,58 |
| 40 | 17,48 | 9,44 | 13,46 | 8,21 | 1,87 |
| 50 | 17,4 | 9,7 | 15,55 | 9,78 | 2,22 |
| 60 | 17,32 | 9,96 | 17,64 | 11,52 | 2,62 |
| 70 | 17,24 | 10,22 | 19,73 | 13,37 | 3,04 |
| 80 | 17,16 | 10,48 | 21,82 | 15,29 | 3,47 |
| 90 | 17,08 | 10,74 | 23,91 | 17,25 | 3,92 |
| 100 | 17 | 11 | 26 | 19,24 | 4,37 |

B) CH + BA

|  | dD | dP | dH | R |  |
| --- | --- | --- | --- | --- | --- |
| Target | 15,9 | 7,7 | 7,3 | 4,8 |  |
| Solvent | dD | dP | dH | Distance | RED |
| Cyclohexanone | 17,8 | 8,4 | 5,1 | 4,45 | 0,93 |
| Benzyl Alcohol | 18,4 | 6,3 | 13,7 | 8,24 | 1,72 |
| % S2 |  |  |  |  |  |
| 0 | 17,8 | 8,4 | 5,1 | 4,45 | 0,93 |
| 10 | 17,86 | 8,19 | 5,96 | 4,17 | 0,87 |
| 20 | 17,92 | 7,98 | 6,82 | 4,08 | 0,85 |
| 30 | 17,98 | 7,77 | 7,68 | 4,18 | 0,87 |
| 40 | 18,04 | 7,56 | 8,54 | 4,46 | 0,93 |
| 50 | 18,1 | 7,35 | 9,4 | 4,89 | 1,02 |
| 60 | 18,16 | 7,14 | 10,26 | 5,43 | 1,13 |
| 70 | 18,22 | 6,93 | 11,12 | 6,06 | 1,26 |
| 80 | 18,28 | 6,72 | 11,98 | 6,75 | 1,41 |
| 90 | 18,34 | 6,51 | 12,84 | 7,48 | 1,56 |
| 100 | 18,4 | 6,3 | 13,7 | 8,24 | 1,72 |

|  | dD | dP | dH | R |  |
| --- | --- | --- | --- | --- | --- |
| Target | 17,2 | 14,6 | 7,1 | 4,4 |  |
| Solvent | dD | dP | dH | Distance | RED |
| Cyclohexanone | 17,8 | 8,4 | 5,1 | 6,62 | 1,51 |
| Benzyl Alcohol | 18,4 | 6,3 | 13,7 | 10,87 | 2,47 |
| % S2 |  |  |  |  |  |
| 0 | 17,8 | 8,4 | 5,1 | 6,62 | 1,51 |
| 10 | 17,86 | 8,19 | 5,96 | 6,64 | 1,51 |
| 20 | 17,92 | 7,98 | 6,82 | 6,78 | 1,54 |
| 30 | 17,98 | 7,77 | 7,68 | 7,03 | 1,60 |
| 40 | 18,04 | 7,56 | 8,54 | 7,38 | 1,68 |
| 50 | 18,1 | 7,35 | 9,4 | 7,82 | 1,78 |
| 60 | 18,16 | 7,14 | 10,26 | 8,33 | 1,89 |
| 70 | 18,22 | 6,93 | 11,12 | 8,90 | 2,02 |
| 80 | 18,28 | 6,72 | 11,98 | 9,52 | 2,16 |
| 90 | 18,34 | 6,51 | 12,84 | 10,18 | 2,31 |
| 100 | 18,4 | 6,3 | 13,7 | 10,87 | 2,47 |

C) CH + Anisol

|  | dD | dP | dH | R |  |
| --- | --- | --- | --- | --- | --- |
| Target | 15,9 | 7,7 | 7,3 | 4,8 |  |
| Solvent | dD | dP | dH | Distance | RED |
| Cyclohexanone | 17,8 | 8,4 | 5,1 | 4,45 | 0,93 |
| Anisole | 17,8 | 4,4 | 6,9 | 5,05 | 1,05 |
| % S2 |  |  |  |  |  |
| 0 | 17,8 | 8,4 | 5,1 | 4,45 | 0,93 |
| 10 | 17,8 | 8 | 5,28 | 4,31 | 0,90 |
| 20 | 17,8 | 7,6 | 5,46 | 4,22 | 0,88 |
| 30 | 17,8 | 7,2 | 5,64 | 4,18 | 0,87 |
| 40 | 17,8 | 6,8 | 5,82 | 4,18 | 0,87 |
| 50 | 17,8 | 6,4 | 6 | 4,22 | 0,88 |
| 60 | 17,8 | 6 | 6,18 | 4,31 | 0,90 |
| 70 | 17,8 | 5,6 | 6,36 | 4,44 | 0,93 |
| 80 | 17,8 | 5,2 | 6,54 | 4,61 | 0,96 |
| 90 | 17,8 | 4,8 | 6,72 | 4,82 | 1,00 |
| 100 | 17,8 | 4,4 | 6,9 | 5,05 | 1,05 |

|  | dD | dP | dH | R |  |
| --- | --- | --- | --- | --- | --- |
| Target | 17,2 | 14,6 | 7,1 | 4,4 |  |
| Solvent | dD | dP | dH | Distance | RED |
| Cyclohexanone | 17,8 | 8,4 | 5,1 | 6,62 | 1,51 |
| Anisole | 17,8 | 4,4 | 6,9 | 10,27 | 2,33 |
| % S2 |  |  |  |  |  |
| 0 | 17,8 | 8,4 | 5,1 | 6,62 | 1,51 |
| 10 | 17,8 | 8 | 5,28 | 6,95 | 1,58 |
| 20 | 17,8 | 7,6 | 5,46 | 7,29 | 1,66 |
| 30 | 17,8 | 7,2 | 5,64 | 7,64 | 1,74 |
| 40 | 17,8 | 6,8 | 5,82 | 7,99 | 1,82 |
| 50 | 17,8 | 6,4 | 6 | 8,36 | 1,90 |
| 60 | 17,8 | 6 | 6,18 | 8,73 | 1,98 |
| 70 | 17,8 | 5,6 | 6,36 | 9,11 | 2,07 |
| 80 | 17,8 | 5,2 | 6,54 | 9,49 | 2,16 |
| 90 | 17,8 | 4,8 | 6,72 | 9,88 | 2,25 |
| 100 | 17,8 | 4,4 | 6,9 | 10,27 | 2,33 |

D) CP + EG

|  | dD | dP | dH | R |  |
| --- | --- | --- | --- | --- | --- |
| Target | 15,9 | 7,7 | 7,3 | 4,8 |  |
| Solvent | dD | dP | dH | Distance | RED |
| Cyclopentanone | 17,9 | 11,9 | 5,2 | 6,17 | 1,29 |
| Ethylene Glycol | 17 | 11 | 26 | 19,12 | 3,98 |
| % S2 |  |  |  |  |  |
| 0 | 17,9 | 11,9 | 5,2 | 6,17 | 1,29 |
| 10 | 17,81 | 11,81 | 7,28 | 5,61 | 1,17 |
| 20 | 17,72 | 11,72 | 9,36 | 5,80 | 1,21 |
| 30 | 17,63 | 11,63 | 11,44 | 6,68 | 1,39 |
| 40 | 17,54 | 11,54 | 13,52 | 8,01 | 1,67 |
| 50 | 17,45 | 11,45 | 15,6 | 9,62 | 2,00 |
| 60 | 17,36 | 11,36 | 17,68 | 11,39 | 2,37 |
| 70 | 17,27 | 11,27 | 19,76 | 13,25 | 2,76 |
| 80 | 17,18 | 11,18 | 21,84 | 15,17 | 3,16 |
| 90 | 17,09 | 11,09 | 23,92 | 17,13 | 3,57 |
| 100 | 17 | 11 | 26 | 19,12 | 3,98 |

|  | dD | dP | dH | R |  |
| --- | --- | --- | --- | --- | --- |
| Target | 17,2 | 14,6 | 7,1 | 4,4 |  |
| Solvent | dD | dP | dH | Distance | RED |
| Cyclopentanone | 17,9 | 11,9 | 5,2 | 3,59 | 0,82 |
| Ethylene Glycol | 17 | 11 | 26 | 19,24 | 4,37 |
| % S2 |  |  |  |  |  |
| 0 | 17,9 | 11,9 | 5,2 | 3,59 | 0,82 |
| 10 | 17,81 | 11,81 | 7,28 | 3,05 | 0,69 |
| 20 | 17,72 | 11,72 | 9,36 | 3,81 | 0,86 |
| 30 | 17,63 | 11,63 | 11,44 | 5,33 | 1,21 |
| 40 | 17,54 | 11,54 | 13,52 | 7,14 | 1,62 |
| 50 | 17,45 | 11,45 | 15,6 | 9,08 | 2,06 |
| 60 | 17,36 | 11,36 | 17,68 | 11,07 | 2,52 |
| 70 | 17,27 | 11,27 | 19,76 | 13,09 | 2,98 |
| 80 | 17,18 | 11,18 | 21,84 | 15,13 | 3,44 |
| 90 | 17,09 | 11,09 | 23,92 | 17,18 | 3,91 |
| 100 | 17 | 11 | 26 | 19,24 | 4,37 |

E) CP + BA

|  | dD | dP | dH | R |  |
| --- | --- | --- | --- | --- | --- |
| Target | 15,9 | 7,7 | 7,3 | 4,8 |  |
| Solvent | dD | dP | dH | Distance | RED |
| Cyclopentanone | 17,9 | 11,9 | 5,2 | 6,17 | 1,29 |
| Benzyl Alcohol | 18,4 | 6,3 | 13,7 | 8,24 | 1,72 |
| % S2 |  |  |  |  |  |
| 0 | 17,9 | 11,9 | 5,2 | 6,17 | 1,29 |
| 10 | 17,95 | 11,34 | 6,05 | 5,62 | 1,17 |
| 20 | 18 | 10,78 | 6,9 | 5,22 | 1,09 |
| 30 | 18,05 | 10,22 | 7,75 | 5,00 | 1,04 |
| 40 | 18,1 | 9,66 | 8,6 | 4,99 | 1,04 |
| 50 | 18,15 | 9,1 | 9,45 | 5,18 | 1,08 |
| 60 | 18,2 | 8,54 | 10,3 | 5,56 | 1,16 |
| 70 | 18,25 | 7,98 | 11,15 | 6,08 | 1,27 |
| 80 | 18,3 | 7,42 | 12 | 6,72 | 1,40 |
| 90 | 18,35 | 6,86 | 12,85 | 7,45 | 1,55 |
| 100 | 18,4 | 6,3 | 13,7 | 8,24 | 1,72 |

|  | dD | dP | dH | R |  |
| --- | --- | --- | --- | --- | --- |
| Target | 17,2 | 14,6 | 7,1 | 4,4 |  |
| Solvent | dD | dP | dH | Distance | RED |
| Cyclopentanone | 17,9 | 11,9 | 5,2 | 3,59 | 0,82 |
| Benzyl Alcohol | 18,4 | 6,3 | 13,7 | 10,87 | 2,47 |
| % S2 |  |  |  |  |  |
| 0 | 17,9 | 11,9 | 5,2 | 3,59 | 0,82 |
| 10 | 17,95 | 11,34 | 6,05 | 3,74 | 0,85 |
| 20 | 18 | 10,78 | 6,9 | 4,15 | 0,94 |
| 30 | 18,05 | 10,22 | 7,75 | 4,74 | 1,08 |
| 40 | 18,1 | 9,66 | 8,6 | 5,47 | 1,24 |
| 50 | 18,15 | 9,1 | 9,45 | 6,28 | 1,43 |
| 60 | 18,2 | 8,54 | 10,3 | 7,14 | 1,62 |
| 70 | 18,25 | 7,98 | 11,15 | 8,04 | 1,83 |
| 80 | 18,3 | 7,42 | 12 | 8,97 | 2,04 |
| 90 | 18,35 | 6,86 | 12,85 | 9,91 | 2,25 |
| 100 | 18,4 | 6,3 | 13,7 | 10,87 | 2,47 |

F) CP + Anisol

|  | dD | dP | dH | R |  |
| --- | --- | --- | --- | --- | --- |
| Target | 15,9 | 7,7 | 7,3 | 4,8 |  |
| Solvent | dD | dP | dH | Distance | RED |
| Cyclopentanone | 17,9 | 11,9 | 5,2 | 6,17 | 1,29 |
| Anisole | 17,8 | 4,4 | 6,9 | 5,05 | 1,05 |
| % S2 |  |  |  |  |  |
| 0 | 17,9 | 11,9 | 5,2 | 6,17 | 1,29 |
| 10 | 17,89 | 11,15 | 5,37 | 5,61 | 1,17 |
| 20 | 17,88 | 10,4 | 5,54 | 5,11 | 1,06 |
| 30 | 17,87 | 9,65 | 5,71 | 4,67 | 0,97 |
| 40 | 17,86 | 8,9 | 5,88 | 4,34 | 0,90 |
| 50 | 17,85 | 8,15 | 6,05 | 4,12 | 0,86 |
| 60 | 17,84 | 7,4 | 6,22 | 4,04 | 0,84 |
| 70 | 17,83 | 6,65 | 6,39 | 4,10 | 0,85 |
| 80 | 17,82 | 5,9 | 6,56 | 4,31 | 0,90 |
| 90 | 17,81 | 5,15 | 6,73 | 4,63 | 0,96 |
| 100 | 17,8 | 4,4 | 6,9 | 5,05 | 1,05 |

|  | dD | dP | dH | R |  |
| --- | --- | --- | --- | --- | --- |
| Target | 17,2 | 14,6 | 7,1 | 4,4 |  |
| Solvent | dD | dP | dH | Distance | RED |
| Cyclopentanone | 17,9 | 11,9 | 5,2 | 3,59 | 0,82 |
| Anisole | 17,8 | 4,4 | 6,9 | 10,27 | 2,33 |
| % S2 |  |  |  |  |  |
| 0 | 17,9 | 11,9 | 5,2 | 3,59 | 0,82 |
| 10 | 17,89 | 11,15 | 5,37 | 4,10 | 0,93 |
| 20 | 17,88 | 10,4 | 5,54 | 4,68 | 1,06 |
| 30 | 17,87 | 9,65 | 5,71 | 5,31 | 1,21 |
| 40 | 17,86 | 8,9 | 5,88 | 5,98 | 1,36 |
| 50 | 17,85 | 8,15 | 6,05 | 6,66 | 1,51 |
| 60 | 17,84 | 7,4 | 6,22 | 7,37 | 1,67 |
| 70 | 17,83 | 6,65 | 6,39 | 8,08 | 1,84 |
| 80 | 17,82 | 5,9 | 6,56 | 8,80 | 2,00 |
| 90 | 17,81 | 5,15 | 6,73 | 9,54 | 2,17 |
| 100 | 17,8 | 4,4 | 6,9 | 10,27 | 2,33 |

G) DMSO + EG

|  | dD | dP | dH | R |  |
| --- | --- | --- | --- | --- | --- |
| Target | 17,2 | 14,6 | 7,1 | 4,4 |  |
| Solvent | dD | dP | dH | Distance | RED |
| DMSO | 18,4 | 16,4 | 10,2 | 4,31 | 0,98 |
| Ethylene glycol | 17,0 | 11,0 | 26,0 | 19,24 | 4,37 |
| % S2 |  |  |  |  |  |
| 0 | 18,4 | 16,4 | 10,2 | 4,31 | 0,98 |
| 10 | 18,26 | 15,86 | 11,78 | 5,29 | 1,20 |
| 20 | 18,12 | 15,32 | 13,36 | 6,56 | 1,49 |
| 30 | 17,98 | 14,78 | 14,94 | 8,00 | 1,82 |
| 40 | 17,84 | 14,24 | 16,52 | 9,51 | 2,16 |
| 50 | 17,7 | 13,7 | 18,1 | 11,08 | 2,52 |
| 60 | 17,56 | 13,16 | 19,68 | 12,68 | 2,88 |
| 70 | 17,42 | 12,62 | 21,26 | 14,30 | 3,25 |
| 80 | 17,28 | 12,08 | 22,84 | 15,94 | 3,62 |
| 90 | 17,14 | 11,54 | 24,42 | 17,59 | 4,00 |
| 100 | 17 | 11 | 26 | 19,24 | 4,37 |

|  | dD | dP | dH | R |  |
| --- | --- | --- | --- | --- | --- |
| Target | 15,9 | 7,7 | 7,3 | 4,8 |  |
| Solvent | dD | dP | dH | Distance | RED |
| DMSO | 18,4 | 16,4 | 10,2 | 10,45 | 2,18 |
| Triethylphosphat | 17,0 | 11,0 | 26,0 | 19,12 | 3,98 |
| % S2 |  |  |  |  |  |
| 0 | 18,4 | 16,4 | 10,2 | 10,45 | 2,18 |
| 10 | 18,26 | 15,86 | 11,78 | 10,44 | 2,17 |
| 20 | 18,12 | 15,32 | 13,36 | 10,70 | 2,23 |
| 30 | 17,98 | 14,78 | 14,94 | 11,22 | 2,34 |
| 40 | 17,84 | 14,24 | 16,52 | 11,95 | 2,49 |
| 50 | 17,7 | 13,7 | 18,1 | 12,87 | 2,68 |
| 60 | 17,56 | 13,16 | 19,68 | 13,93 | 2,90 |
| 70 | 17,42 | 12,62 | 21,26 | 15,11 | 3,15 |
| 80 | 17,28 | 12,08 | 22,84 | 16,38 | 3,41 |
| 90 | 17,14 | 11,54 | 24,42 | 17,72 | 3,69 |
| 100 | 17 | 11 | 26 | 19,12 | 3,98 |

H) DMSO + BA

|  | dD | dP | dH | R |  |
| --- | --- | --- | --- | --- | --- |
| Target | 15,9 | 7,7 | 7,3 | 4,8 |  |
| Solvent | dD | dP | dH | Distance | RED |
| DMSO | 18,4 | 16,4 | 10,2 | 10,45 | 2,18 |
| Benzyl Alcohol | 18,4 | 6,3 | 13,7 | 8,24 | 1,72 |
| % S2 |  |  |  |  |  |
| 0 | 18,4 | 16,4 | 10,2 | 10,45 | 2,18 |
| 10 | 18,4 | 15,39 | 10,55 | 9,73 | 2,03 |
| 20 | 18,4 | 14,38 | 10,9 | 9,09 | 1,89 |
| 30 | 18,4 | 13,37 | 11,25 | 8,53 | 1,78 |
| 40 | 18,4 | 12,36 | 11,6 | 8,07 | 1,68 |
| 50 | 18,4 | 11,35 | 11,95 | 7,74 | 1,61 |
| 60 | 18,4 | 10,34 | 12,3 | 7,55 | 1,57 |
| 70 | 18,4 | 9,33 | 12,65 | 7,50 | 1,56 |
| 80 | 18,4 | 8,32 | 13 | 7,61 | 1,58 |
| 90 | 18,4 | 7,31 | 13,35 | 7,86 | 1,64 |
| 100 | 18,4 | 6,3 | 13,7 | 8,24 | 1,72 |

|  | dD | dP | dH | R |  |
| --- | --- | --- | --- | --- | --- |
| Target | 17,2 | 14,6 | 7,1 | 4,4 |  |
| Solvent | dD | dP | dH | Distance | RED |
| DMSO | 18,4 | 16,4 | 10,2 | 4,31 | 0,98 |
| Benzyl Alcohol | 18,4 | 6,3 | 13,7 | 10,87 | 2,47 |
| % S2 |  |  |  |  |  |
| 0 | 18,4 | 16,4 | 10,2 | 4,31 | 0,98 |
| 10 | 18,4 | 15,39 | 10,55 | 4,28 | 0,97 |
| 20 | 18,4 | 14,38 | 10,9 | 4,50 | 1,02 |
| 30 | 18,4 | 13,37 | 11,25 | 4,95 | 1,12 |
| 40 | 18,4 | 12,36 | 11,6 | 5,57 | 1,27 |
| 50 | 18,4 | 11,35 | 11,95 | 6,31 | 1,43 |
| 60 | 18,4 | 10,34 | 12,3 | 7,14 | 1,62 |
| 70 | 18,4 | 9,33 | 12,65 | 8,02 | 1,82 |
| 80 | 18,4 | 8,32 | 13 | 8,94 | 2,03 |
| 90 | 18,4 | 7,31 | 13,35 | 9,90 | 2,25 |
| 100 | 18,4 | 6,3 | 13,7 | 10,87 | 2,47 |

I) DMSO + Anisol

|  | dD | dP | dH | R |  |
| --- | --- | --- | --- | --- | --- |
| Target | 15,9 | 7,7 | 7,3 | 4,8 |  |
| Solvent | dD | dP | dH | Distance | RED |
| Dimethyl Sulfoxide (DMSO) | 18,4 | 16,4 | 10,2 | 10,45 | 2,18 |
| Anisole | 17,8 | 4,4 | 6,9 | 5,05 | 1,05 |
| % S2 |  |  |  |  |  |
| 0 | 18,4 | 16,4 | 10,2 | 10,45 | 2,18 |
| 10 | 18,34 | 15,2 | 9,87 | 9,31 | 1,94 |
| 20 | 18,28 | 14 | 9,54 | 8,21 | 1,71 |
| 30 | 18,22 | 12,8 | 9,21 | 7,15 | 1,49 |
| 40 | 18,16 | 11,6 | 8,88 | 6,18 | 1,29 |
| 50 | 18,1 | 10,4 | 8,55 | 5,31 | 1,11 |
| 60 | 18,04 | 9,2 | 8,22 | 4,63 | 0,96 |
| 70 | 17,98 | 8 | 7,89 | 4,21 | 0,88 |
| 80 | 17,92 | 6,8 | 7,56 | 4,15 | 0,86 |
| 90 | 17,86 | 5,6 | 7,23 | 4,45 | 0,93 |
| 100 | 17,8 | 4,4 | 6,9 | 5,05 | 1,05 |

|  | dD | dP | dH | R |  |
| --- | --- | --- | --- | --- | --- |
| Target | 17,2 | 14,6 | 7,1 | 4,4 |  |
| Solvent | dD | dP | dH | Distance | RED |
| Dimethyl Sulfoxide (DMSO) | 18,4 | 16,4 | 10,2 | 4,31 | 0,98 |
| Anisole | 17,8 | 4,4 | 6,9 | 10,27 | 2,33 |
| % S2 |  |  |  |  |  |
| 0 | 18,4 | 16,4 | 10,2 | 4,31 | 0,98 |
| 10 | 18,34 | 15,2 | 9,87 | 3,64 | 0,83 |
| 20 | 18,28 | 14 | 9,54 | 3,31 | 0,75 |
| 30 | 18,22 | 12,8 | 9,21 | 3,44 | 0,78 |
| 40 | 18,16 | 11,6 | 8,88 | 3,98 | 0,90 |
| 50 | 18,1 | 10,4 | 8,55 | 4,79 | 1,09 |
| 60 | 18,04 | 9,2 | 8,22 | 5,77 | 1,31 |
| 70 | 17,98 | 8 | 7,89 | 6,83 | 1,55 |
| 80 | 17,92 | 6,8 | 7,56 | 7,95 | 1,81 |
| 90 | 17,86 | 5,6 | 7,23 | 9,10 | 2,07 |
| 100 | 17,8 | 4,4 | 6,9 | 10,27 | 2,33 |

J) TEP + EG

|  | dD | dP | dH | R |  |
| --- | --- | --- | --- | --- | --- |
| Target | 15,9 | 7,7 | 7,3 | 4,8 |  |
| Solvent | dD | dP | dH | Distance | RED |
| Triethylphosphate | 16,7 | 11,4 | 9,2 | 4,46 | 0,93 |
| Ethylene Glycol | 17 | 11 | 26 | 19,12 | 3,98 |
| % S2 |  |  |  |  |  |
| 0 | 16,7 | 11,4 | 9,2 | 4,46 | 0,93 |
| 10 | 16,73 | 11,36 | 10,88 | 5,38 | 1,12 |
| 20 | 16,76 | 11,32 | 12,56 | 6,61 | 1,38 |
| 30 | 16,79 | 11,28 | 14,24 | 8,01 | 1,67 |
| 40 | 16,82 | 11,24 | 15,92 | 9,50 | 1,98 |
| 50 | 16,85 | 11,2 | 17,6 | 11,04 | 2,30 |
| 60 | 16,88 | 11,16 | 19,28 | 12,62 | 2,63 |
| 70 | 16,91 | 11,12 | 20,96 | 14,23 | 2,96 |
| 80 | 16,94 | 11,08 | 22,64 | 15,85 | 3,30 |
| 90 | 16,97 | 11,04 | 24,32 | 17,48 | 3,64 |
| 100 | 17 | 11 | 26 | 19,12 | 3,98 |

|  | dD | dP | dH | R |  |
| --- | --- | --- | --- | --- | --- |
| Target | 17,2 | 14,6 | 7,1 | 4,4 |  |
| Solvent | dD | dP | dH | Distance | RED |
| Triethylphosphate | 16,7 | 11,4 | 9,2 | 3,96 | 0,90 |
| Ethylene Glycol | 17 | 11 | 26 | 19,24 | 4,37 |
| % S2 |  |  |  |  |  |
| 0 | 16,7 | 11,4 | 9,2 | 3,96 | 0,90 |
| 10 | 16,73 | 11,36 | 10,88 | 5,07 | 1,15 |
| 20 | 16,76 | 11,32 | 12,56 | 6,43 | 1,46 |
| 30 | 16,79 | 11,28 | 14,24 | 7,92 | 1,80 |
| 40 | 16,82 | 11,24 | 15,92 | 9,47 | 2,15 |
| 50 | 16,85 | 11,2 | 17,6 | 11,06 | 2,51 |
| 60 | 16,88 | 11,16 | 19,28 | 12,67 | 2,88 |
| 70 | 16,91 | 11,12 | 20,96 | 14,30 | 3,25 |
| 80 | 16,94 | 11,08 | 22,64 | 15,94 | 3,62 |
| 90 | 16,97 | 11,04 | 24,32 | 17,59 | 4,00 |
| 100 | 17 | 11 | 26 | 19,24 | 4,37 |

K) TEP + BA

|  | dD | dP | dH | R |  |
| --- | --- | --- | --- | --- | --- |
| Target | 15,9 | 7,7 | 7,3 | 4,8 |  |
| Solvent | dD | dP | dH | Distance | RED |
| Triethylphosphate | 16,7 | 11,4 | 9,2 | 4,46 | 0,93 |
| Benzyl Alcohol | 18,4 | 6,3 | 13,7 | 8,24 | 1,72 |
| % S2 |  |  |  |  |  |
| 0 | 16,7 | 11,4 | 9,2 | 4,46 | 0,93 |
| 10 | 16,87 | 10,89 | 9,65 | 4,41 | 0,92 |
| 20 | 17,04 | 10,38 | 10,1 | 4,50 | 0,94 |
| 30 | 17,21 | 9,87 | 10,55 | 4,70 | 0,98 |
| 40 | 17,38 | 9,36 | 11 | 5,02 | 1,05 |
| 50 | 17,55 | 8,85 | 11,45 | 5,43 | 1,13 |
| 60 | 17,72 | 8,34 | 11,9 | 5,90 | 1,23 |
| 70 | 17,89 | 7,83 | 12,35 | 6,43 | 1,34 |
| 80 | 18,06 | 7,32 | 12,8 | 7,00 | 1,46 |
| 90 | 18,23 | 6,81 | 13,25 | 7,61 | 1,59 |
| 100 | 18,4 | 6,3 | 13,7 | 8,24 | 1,72 |

|  | dD | dP | dH | R |  |
| --- | --- | --- | --- | --- | --- |
| Target | 17,2 | 14,6 | 7,1 | 4,4 |  |
| Solvent | dD | dP | dH | Distance | RED |
| Triethylphosphate | 16,7 | 11,4 | 9,2 | 3,96 | 0,90 |
| Benzyl Alcohol | 18,4 | 6,3 | 13,7 | 10,87 | 2,47 |
| % S2 |  |  |  |  |  |
| 0 | 16,7 | 11,4 | 9,2 | 3,96 | 0,90 |
| 10 | 16,87 | 10,89 | 9,65 | 4,55 | 1,03 |
| 20 | 17,04 | 10,38 | 10,1 | 5,19 | 1,18 |
| 30 | 17,21 | 9,87 | 10,55 | 5,85 | 1,33 |
| 40 | 17,38 | 9,36 | 11 | 6,54 | 1,49 |
| 50 | 17,55 | 8,85 | 11,45 | 7,24 | 1,65 |
| 60 | 17,72 | 8,34 | 11,9 | 7,96 | 1,81 |
| 70 | 17,89 | 7,83 | 12,35 | 8,68 | 1,97 |
| 80 | 18,06 | 7,32 | 12,8 | 9,40 | 2,14 |
| 90 | 18,23 | 6,81 | 13,25 | 10,14 | 2,30 |
| 100 | 18,4 | 6,3 | 13,7 | 10,87 | 2,47 |

L) TEP + Anisol

|  | dD | dP | dH | R |  |
| --- | --- | --- | --- | --- | --- |
| Target | 15,9 | 7,7 | 7,3 | 4,8 |  |
| Solvent | dD | dP | dH | Distance | RED |
| Triethylphosphate | 16,7 | 11,4 | 9,2 | 4,46 | 0,93 |
| Anisole | 17,8 | 4,4 | 6,9 | 5,05 | 1,05 |
| % S2 |  |  |  |  |  |
| 0 | 16,7 | 11,4 | 9,2 | 4,46 | 0,93 |
| 10 | 16,81 | 10,7 | 8,97 | 3,89 | 0,81 |
| 20 | 16,92 | 10 | 8,74 | 3,39 | 0,71 |
| 30 | 17,03 | 9,3 | 8,51 | 3,02 | 0,63 |
| 40 | 17,14 | 8,6 | 8,28 | 2,81 | 0,59 |
| 50 | 17,25 | 7,9 | 8,05 | 2,81 | 0,59 |
| 60 | 17,36 | 7,2 | 7,82 | 3,01 | 0,63 |
| 70 | 17,47 | 6,5 | 7,59 | 3,37 | 0,70 |
| 80 | 17,58 | 5,8 | 7,36 | 3,86 | 0,80 |
| 90 | 17,69 | 5,1 | 7,13 | 4,43 | 0,92 |
| 100 | 17,8 | 4,4 | 6,9 | 5,05 | 1,05 |

|  | dD | dP | dH | R |  |
| --- | --- | --- | --- | --- | --- |
| Target | 17,2 | 14,6 | 7,1 | 4,4 |  |
| Solvent | dD | dP | dH | Distance | RED |
| Triethylphosphate | 16,7 | 11,4 | 9,2 | 3,96 | 0,90 |
| Anisole | 17,8 | 4,4 | 6,9 | 10,27 | 2,33 |
| % S2 |  |  |  |  |  |
| 0 | 16,7 | 11,4 | 9,2 | 3,96 | 0,90 |
| 10 | 16,81 | 10,7 | 8,97 | 4,39 | 1,00 |
| 20 | 16,92 | 10 | 8,74 | 4,92 | 1,12 |
| 30 | 17,03 | 9,3 | 8,51 | 5,49 | 1,25 |
| 40 | 17,14 | 8,6 | 8,28 | 6,12 | 1,39 |
| 50 | 17,25 | 7,9 | 8,05 | 6,77 | 1,54 |
| 60 | 17,36 | 7,2 | 7,82 | 7,44 | 1,69 |
| 70 | 17,47 | 6,5 | 7,59 | 8,13 | 1,85 |
| 80 | 17,58 | 5,8 | 7,36 | 8,84 | 2,01 |
| 90 | 17,69 | 5,1 | 7,13 | 9,55 | 2,17 |
| 100 | 17,8 | 4,4 | 6,9 | 10,27 | 2,33 |

| Table S4. Solubility according to Hansen approach. | | | |
| --- | --- | --- | --- |
| mixture | solubility according to Hansen prediction  [% non-solvent] | selected mixtures for the phase inversion process | experimental solubility (12 wt.% PVdF-HFP) at 70 °C; up to 50 wt.% non-solvent was investigated [% non-solvent] |
| CH + EG | 0 – 20 |  |  |
| CH + BA | 0 – 40 |  | 0 – 20 |
| CH + An | 0 – 80 |  | 0 – 30 |
| CP + EG | 0 – 20 | x | 0 – 20 |
| CP + BA | 0 – 20 | x | 0 – 20 |
| CP + An | 0 – 10 / 30 – 90 |  |  |
| DMSO + EG | 0 |  |  |
| DMSO + BA | 0 – 10 |  | 0 – 30 |
| DMSO + An | 0 – 40 |  | 0 – 50 |
| TEP + EG | 0 |  |  |
| TEP + BA | 0 – 30 |  | 0 – 30 |
| TEP + An | 0 – 90 |  |  |

**References**

[1] C.M. Hansen, Hansen solubility parameters, a user's handbook, CRC Press2007.

[2] S. Abbott, H. Yamamoto, HSPiP, software, 5th Ed. (2015).
